# Nevers City Earthenware Blue Glaze: pXRF Categorization from Cobalt Sources and Raw Materials Impurities: Comparison of Reasoned and Chemometrics Methods

**DOI:** 10.3390/ma19122442

**Published:** 2026-06-07

**Authors:** Gulsu Simsek-Franci, Philippe Colomban, Marie-Lys Chevalier

**Affiliations:** 1 Department of Metallurgical and Materials Engineering, Faculty of Chemical and Metallurgical Engineering, Yildiz Technical University, Davutpasa Mah. Davutpasa Caddesi, 34220 Istanbul, Türkiye; gulsu.simsek@yildiz.edu.tr; 2 Laboratoire ‘De la Molécule au Nano-Objet: Réactivité, Interaction et Spectroscopies, (MONARIS UMR8233), Sorbonne Université, CNRS, Campus P.-et-M. Curie, 4 Place Jussieu, 75005 Paris, France; 3 Musée de la Faïence et des Beaux-Arts-‘Frédéric Blandin’, 16 rue Saint-Genest, 58000 Nevers, France

**Keywords:** earthenware, glaze, Nevers, pXRF, 17th century, 18th century, 19th century, cobalt, composition, Naples yellow, white, blue

## Abstract

The blue, white, and black glazed areas of nineteen Nevers earthenware pieces bearing a date or precisely datable between 1589 and 1865 were for the first time analyzed by X-ray fluorescence spectroscopy at the Musée de la faïence et des Beaux-Arts-‘Frédéric Blandin’ in the city of Nevers by pXRF in order to categorize the raw materials and recipes used. The semi-quantitative signal comparison of major elements and impurities such as rubidium, strontium and zirconium shows the use of the same raw materials except for six artifacts. At least three types of cobalt, characterized by association with copper, nickel, and manganese, are observed. Different blacks (with manganese or bismuth) are observed. A comparison is made between the classification obtained with chemometry (z-score, PCA, and dendrograms of similarity) and a reasoned analysis of ternary diagrams based on the signal of the most characteristic elements. This preliminary work demonstrates the potential provided for the categorization of enameled ceramics and their dating through non-invasive on-site semi-quantitative elemental analyses. No important advantages were observed for the chemometric procedure: the same conclusions are obtained by quantitative comparisons of the XRF data, but the chemometric procedures allow a clear visualization of the main conclusions.

## 1. Introduction

The production of polychrome earthenware coated with a tin-opacified glaze, i.e., faience, in Nevers appeared in the middle of the 16th century and, together with those of Rouen and Lyon, constituted the beginning of this type of pottery in France. Many (Italian) potters first established themselves in Lyon before moving to Nevers. The last historic factory ceased its activity at the end of the 20th century, while the Rouen factories disappeared under Napoleon III, as did the Lyon factories. Nevers faience therefore occupies an important place in French production. However, few technical studies have been devoted to it. The four volumes by J. Rosen published in 2009 provide the state of the art of historical and stylistic knowledge of production until the end of the 19th century and give some basic analyses of the pastes obtained on potsherds by invasive methods [1]. The use of iron oxides to obtain red has also been studied [2,3], but no work has been done concerning the nature of the white and blue glaze, especially regarding “cobalt”, nor concerning the different yellow and black pigments. Even for other major European centers of earthenware production such as Delft, the work is limited and concerns almost only the paste [4,5,6,7]. There is no in-depth work carried out with modern techniques for similar earthenware like Rouen, Moustiers, Marseille, etc., wares. The famous dishes of Bernard Palissy have been studied [8], along with the Portuguese azulejos (e.g., [9,10] and references herein) and productions of lower renown (Switzerland, Slovenia, etc.) [4,11] or finds from outside Europe [12].

In the non-invasive study of Islamic pottery (Minai [13], Timurid [14], Ottoman [14,15,16], Safavid [14] and Hafsid [17]), most of which are coated with tin and lead-containing glaze, and European [18,19] and Asian [20,21] porcelain also coated with more complex glaze, we have shown that non-invasive, on-site semi-quantitative X-ray fluorescence spectroscopic (XRF) study of the chemical elements present in glazes makes it possible to follow developments in production processes and selection of raw materials. This is particularly true for the blue coloring agent Co^2+^ ions due to the rarity of this element, especially in deposits sufficiently rich to be exploited [22,23,24,25,26,27]. In fact, cobalt was—and continues to be—a by-product of the mining of other elements such as silver, copper, bismuth, zinc, and arsenic [23,24,25,26,27]. During the European Middle Ages, Renaissance, up to the 18th century, the main European sources were veins exploited for silver (coinage and jewelry) and bismuth (lead hardening for casting printing types in great development after Gutenberg’s printing innovation), the so-called “Five element” veins [22,27]. Another main use of cobalt was for whitening linen fabrics [28], paper [29], and a pigment for oil and fresco paintings and for glass and glaze coloring [30,31].

The present work is based on the reasoned quantitative comparison of the areas of X-ray fluorescence peaks [18,19,20,21,22], which is more informative than the simple identification of associated elements, as made since the pioneering work of Kaczmarczyk in 1986 [32], by Gratuze et al. in 1992 and 1996 [33,34], Porter in 1997 [35] and Delamare in the 2000s [36] for different glass and glaze from the Mediterranean and Islamic worlds. We will also compare this “reasoned” classification with that made using chemometrics (z-score, principal component analysis and Euclidean hierarchical similarity distances).

The final composition of coloring areas obtained by adding coloring agents to the colorless glaze also depends on chemical, physical, and thermal treatment of the ores. Roughly speaking, until the 17th century the preparation of coloring materials was basic, mainly involving visual selection of the appropriate “ore” fragment, grinding and washing, roasting and basic chemical treatments (acids and washing), final thermal treatment (eventually mixing with glass as in smalt) and grinding/sieving [27,37,38,39,40,41,42,43,44]. Refining treatments improved in the 18th century, but it was in the 19th century, mainly after 1850, that the use of “pure” chemicals (sulphates, carbonates, nitrates, and even transition metal oxides) became common in glaze production. It follows that for a long-time, significant quantities of chemical elements other than the chromophore element, characteristic of the geological/geochemical context of the mine and not eliminated during “purification”, remain present in the colored glaze. This is particularly true for cobalt, a rare element with rare deposits capable of being exploited technically and economically, as a by-product of the exploitation of silver, copper, arsenic, and bismuth depending on place and time [22,23,24,25,26,27]. Furthermore, cobalt is generally added in the form of smalt, a cobalt-rich potash glass prepared from the cobalt-rich slag (saffre) obtained in the preparation of silver or bismuth [27,37,38,39,40,41,42,43,44]. These elements will serve as classification parameters.

## 2. Method and Materials

### 2.1. X-Ray Fluorescence Spectroscopy

**Figure 1:**
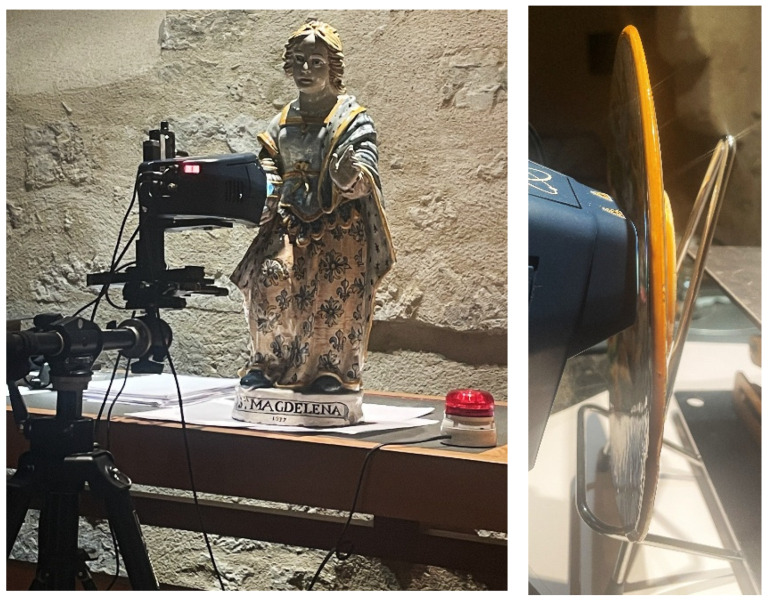
View of the XRF spectrometer mounted on a photographic tripod via a motorized X-Y stage, the movement perpendicular to the object (Z) being manually controlled by a micrometric device. The detailed view shows the absence of contact with the object.

The analytical protocol follows methods described in previous studies [13,14,15,16,17,18,19,20,21]. XRF analysis was performed using a portable Elio instrument (Bruker, Berlin, Germany) featuring a rhodium-anode X-ray tube, a ~1 mm^2^ collimator, and a large-area Silicon Drift Detector (detection range: 1.3–43 keV in air; energy resolution < 140 eV for Mn Kα transition). The instrument was mounted on a dedicated motorized frame to ensure stable focusing, and measurements were taken in point mode for 180 s (360 s for blue areas) at 50 kV and 80 μA, with no filter between tube and sample (Figure 1). Peak energies of characteristic element K, L and M transitions are tabulated [45]. The analysis depth, estimated using the Beer–Lambert law (defined as the layer from which 90% of the fluorescence originates), was approximately 6 μm for Si Kα, 170 μm for Cu Kα, and 300 μm for Au Lα—comparable to glaze thicknesses—but 3 mm for Sn Kα [46]. Accuracy was verified using reference glass and stone materials. All the recorded spectra are given in Figure S1 (Supplementary Materials).

Since the intensity of electronic transition peaks (Kα, Kβ, Mα, Mβ, Lα, Lβ, Lγ, etc.) depends on both elemental identity, absorption and concentration, peaks for trace elements like Pb and Rb can appear more intense than those for major components like Si or Al. Due to topological variations in the 3D distribution of colorants within glazed coatings of variable thickness, calculating a “composition” from surface measurements is not meaningful. Instead, a clustering analysis method developed by our group was used [13,14,15,16,17,18,19,20,21,27]. This involved plotting normalized peak areas (calculated with Artax 7.4.0.0, Bruker AXS GmbH, Berlin, Germany, see Figure S2 (Supplementary Materials)) in ternary and biplot scatter diagrams. Normalization was performed relative to the Rh tube peak, and for blue-decorated samples, also relative to the Co or Si signal for highlighting signal comparison. The normalized data were then plotted in ternary scatter plots for interpretation and discussion using Statistica^®^ 13.5.0.17. Since the 1980s, chemometrics, also known in the archaeometry literature as multivariate statistical analysis [47,48,49,50,51,52,53,54,55,56,57,58,59,60], has been particularly well suited to the analysis of complex and high-dimensional data, such as semi-quantitative pXRF analyses, enabling the extraction of meaningful patterns and features from intricate datasets. Today, even social scientists without a strong background in mathematics or statistics can use software such as Statistica^®^ 14.1.0 developed by TIBCO Software (Palo Alto, CA, USA), analytical modules developed by the Python Software Foundation (Wilmington, DE, USA), Minitab^®^ 22 (State College, Pennsylvania, PA, USA), and SPSS^®^ v30 developed by IBM (Armonk, NY, USA) to compare datasets and make various technological interpretations. For clustering/similarity analysis (dendrogram), the dataset was first explored using the Pandas 3.0.x (NumFOCUS, Inc., Austin, TX, USA) library. Agglomerative clustering from the sklearn.cluster^®^ 1.9.0 module was then performed. To visualize and illustrate the composition of each cluster, dendrograms were generated, displaying the points in each cluster and their relative distances. Ward’s method was selected as the linkage method, implemented using the scipy.cluster.hierarchy^®^ 1.17.1 module (open source, GitHub, Inc., San Francisco, CA, USA) for visual representation and matplotlib^®^ 3.10.9 for plotting. The code was developed and executed in a Jupyter Lab^®^ environment, version 4.1.6 (Jupyter.org, Santa Teresa, NM, USA).

One of the primary aims of using geochemistry in exploration is to identify anomalies in certain elements that may indicate the presence of specific mineral deposits or purification treatments applied to raw materials prior to production. Reliable assessments of these anomalies are best achieved through statistical techniques. These include parametric measurements of central tendency and dispersion, such as the mean, standard deviation, and variance.

The z-score, a standardization method, is defined as the distance of an observational measurement, *x*, from the mean in units of standard deviation [61]. In this study, it was calculated by subtracting the mean and dividing by the standard deviation for the peak areas of each element. This transformation results in each element having a mean of 0 and a standard deviation of 1, thereby simplifying the identification of anomalous values [62,63]. As stated in Brereton’s recent review [61], standardization of the columns representing the analytical data is a common transformation prior to principal component analysis (PCA).

Since ternary scatter plots require non-negative values representing proportional relationships, the calculated z-scores were further transformed using the Cumulative Distribution Function (CDF) of the standard normal distribution [62]. This sigmoid-like transformation maps the unbounded z-score values onto a constrained interval of [0, 1]. By applying this function, the statistical significance of each element’s variation was preserved while ensuring a valid coordinate system for ternary representation [63]. This step enabled the visualization of relative elemental enrichments (positive z-scores) and depletions (negative z-scores) within a unified triangular space.

In this study, we aimed to compare clusters, dendrograms, and PCA obtained from Rh-normalized peak areas with those derived from the z-score transformation after Rh normalization. This approach prevents dominant major elements from overshadowing trace elements, which may contain more diagnostically relevant information to distinguish different uses of raw materials, as well as different production technologies.

### 2.2. Objects

Table 1 presents the 19 selected objects. They were selected based on the existence of a date or historical elements allowing their dating among the four periods identified by J. Rosen in his stylistic study [1]: the first century marked by the inspiration of Italian majolica (1585–1660); the second period in the so-called “Persian” (1630–1700), Chinese (1650–1750), and Franco-Nivernais (1635–1789) tastes; the third period of more eclectic inspiration following the design of Rouen and Moustier earthenware (1700–1789) or Saxony porcelain (1770–1789); and the last period with “Revolution” thematic decorations and a certain technical decadence (1789–1900). Some historical information is given below for the most characteristic artifacts.

The two oldest analyzed pieces (a tile depicting initials and a flower) belong to a panel of 15 tiles from the floor of the Ducal Palace of Nevers (inv. NF 32B, 77.5 × 50 cm^2^, each tile approximately 14.5 cm on a side) decorated with various emblems of the Dukes of Gonzagues, attributed to Augustin Conrade and Jules Gambin, around 1588–1589. This castle is considered the first of the “Châteaux de la Loire” to have been built. Conrade was from Albisola in Liguria. After spending a year in Lyon (1577), he joined Nevers in 1585 with another Italian, Pierre Pertuis (Pertini), and associated with the noble Masters of the Glassworks of Nevers to create the first majolica workshop in Nevers. The tiles analyzed are a tile decorated with four intertwined C characters (Figure 53, T2, ref. [1]) and a tile decorated with a flower. The four intertwined C characters are interpreted as the initials of Charles de Gonzague-Clèves, the first (future) duke born in Nevers in 1580.

The circular Italian-style plate (diameter: 23 cm), circa 1640 (inv. NF 97121), from a series marked in cursive “*Gallateé. co gliamore*” presenting the same setting of mountains and city but with different mythological scenes, here “*Scylla and Glaucus*” (Figure 233, T2 in ref. [1]), inspired by an engraving by Bernard Salomon for “*The Metamorphosis of Ovid*” (Jean de Tournes, Lyon, 1557) is decorated following the famous style of the *istoriato* majolica of Urbino and other Italian cities.

The statue of Saint Madeleine *myrophore* (inv. NF 1726, h: 66 cm), decor *a compendiaro*, blue dress and *fleur de lys*, with the inscription *Sta Magdelena 1637* on the base, is representative of the Franco-Nivernaise period like the workshop sign (inv. MNC 8330, deposit of the Musée national de Céramique, Sèvres, 35 × 35 cm^2^) of an earthenware merchant, decor *a compendiaro* of two pastoral characters around a flowered vase and inscription “*CEANS SE FAICT ET VENDS/DE TOUTE SORTE DE FA/IANCE 1658*”.

The statuette of a water bearer (inv. NF 1677, h: 26 cm, deposit of the Musée Carnavalet), with a blue background with a dispersion of white “candle” splashes, circa 1660–1680, is characteristic of so-called “Persian” productions.

The salad bowl with contoured edges and oblique gadroons (MNC 27838, d: 31.3 cm), with blue-and-white decoration, “*à l’arbre d’amours*” decoration, inscription “*Jacques Cretai Et marie: trotos Femme de jacques/Cretai 1765*”, attributed to C.G. Bigourat (Figure 398, T3 in ref. [1]) is one of the large dishes imitating blue-and-white Ming porcelain, very popular at the time. The same is true of the circular dish (inv. NF242, d: 47.6 cm), blue monochrome decoration, “*The appearance of Christ to a saint*”, reverse with stylized motifs and a large flower, dated 1665.

The goblet on a pedestal (inv. NF 860, h: 15.5 cm), polychrome, reserve on a speckled background, inscriptions *1792*, *Serizier* and *l’an 4/de la Liberté* is an example of so-called “revolutionary” earthenware prized by French collectors; Serizier was a faience manufacturer from Nevers (Figure 646, T3, in ref. [1]).

The *calotte* plate (inv. NF828, d: 22 cm), “*à la giraffe*” decoration, circa 1828, relates to the arrival of the giraffe “Zafara” in 1826, offered to the king of France Charles X by the pasha of Egypt, Mehmet-Ali.

The lid of the armorial pot (inv. NF 4, h: 35.5 cm) is a restoration dating to 1865, a period where pure synthetic coloring raw materials became available.

## 3. Results

The spectra obtained for each object and corresponding peak area table are given in Supplementary Materials (Figure S1). The colored areas studied are listed in Table 1. The net number of photons calculated using the software ARTAX is given in Table S1. We will compare the blue and white areas first.

### 3.1. Main Ingredients, Cobalt and Associated Elements: Global View

**Figure 2:**
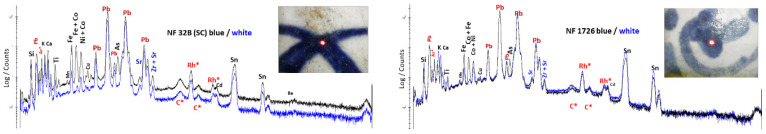

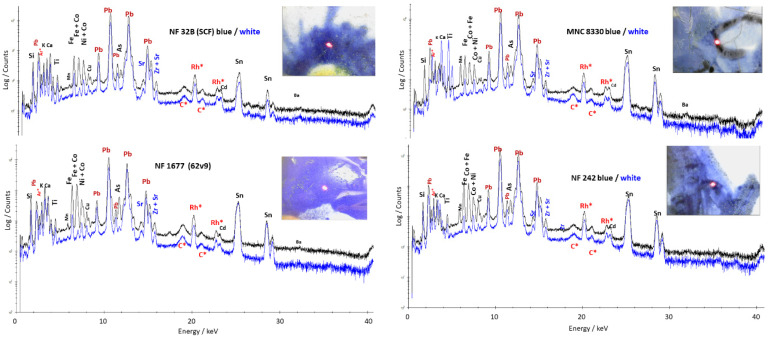
Comparison of XRF spectra recorded on blue and white areas for 17th century objects (see Table 1). Peaks labeled with a star arise from the rhodium source (Rh), argon in air (Ar) or from the Compton Effect (C*).

**Figure 3:**
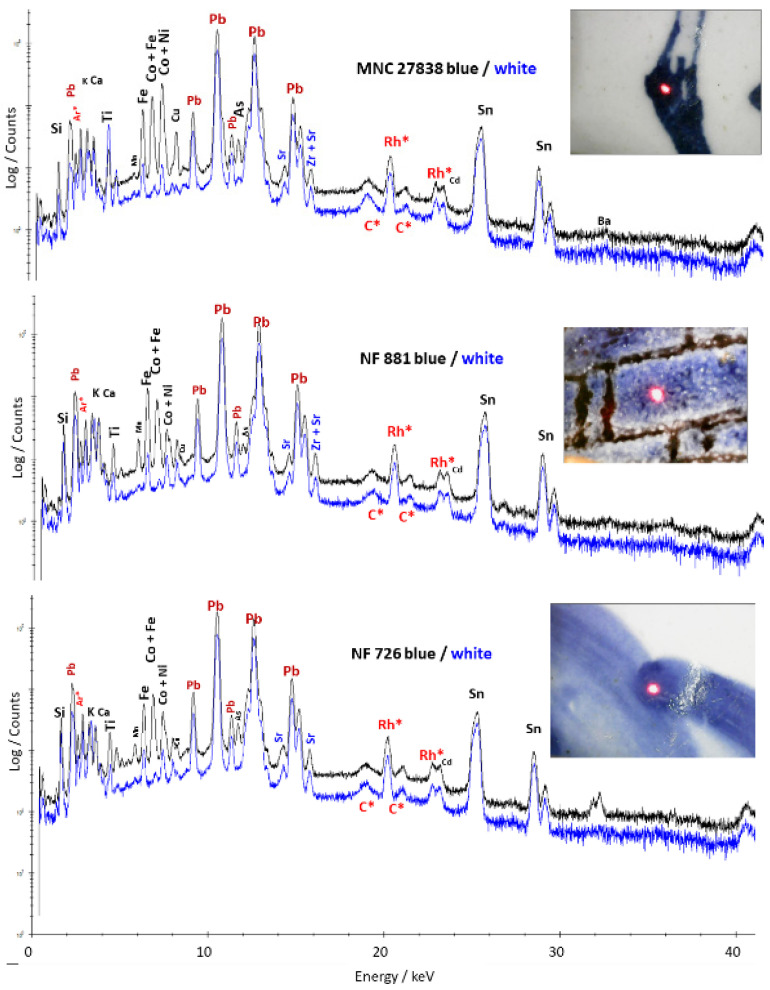
Comparison of XRF spectra recorded on blue and white areas for 18th century objects (see Table 1).

**Figure 4:**
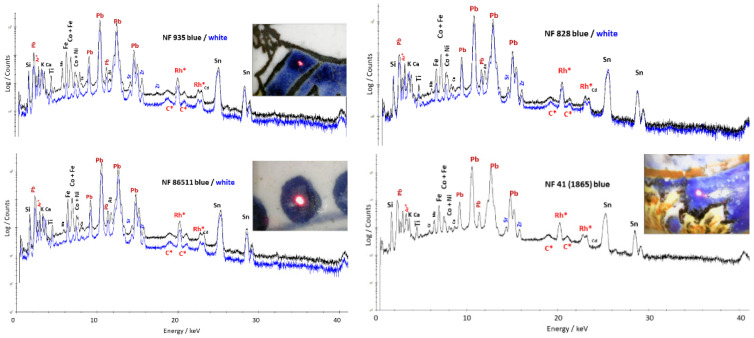
Comparison of XRF spectra recorded on blue and white areas for 19th century objects (see Table 1 for details).

Figure 2, Figure 3 and Figure 4 compare the spectra (logarithmic scale) obtained on the blue area to those obtained on the neighboring white area, respectively, for objects from the 17th, 18th and 19th centuries. To better highlight the differences visually, the counting time for measurements (and hence the peak intensity) on the white background is half as much (except for NF 1726).

The peaks of the Kα and Kβ transitions of cobalt are at 6.93 and 7.65 keV [45], that is to say that the first peak is almost identical to that of the Kβ transition of iron (7.06 keV) for the resolution of the instrument (0.14 keV) in this spectral range. The coloring power of cobalt being exceptional, the content usually ranges between 0.1 and 0.5% wt CoO [27]. Often the presence of cobalt is manifested only by a broadening of the peak towards 7 keV. However, for all the spectra in Figure 2, the increase in the intensity of the peak around 7 keV is evident, and that of the Co Kβ peak associated with 7.65 [45] keV is visible. Thus, we can expect that the cobalt content is close to 0.5% wt or higher. The other peaks having the same behavior are those of arsenic (the Kα peak at 10.54 keV being confused with the Lα of lead at 10.55 keV and only Kβ at 11.73 keV can be observed visually). On the contrary, main peaks of titanium (Kα at 4.51 keV), copper (Kα at 8.05 keV), nickel (Kα at 7.48 keV), manganese (Kα at 5.9 keV) and chromium (Kα at 5.41 keV) are almost free of overlapping.

The same conclusions can be made for Figure 3 (18th century objects), but the intensities of the Kα peaks of arsenic and copper appear less intense than for Figure 2 (17th century). The same is true for Figure 4, comparing the spectra of 19th-century objects. For the object dating from 1865 (lid made in 1865 for the NF 41 pot), arsenic no longer appears; copper is minimal; and the detection of traces of manganese and chromium comes from the proximity of a black line which contributes to the spectrum.

Some other small peaks can be identified: for instance, cadmium at 23.17 (Kα) and 26.10 (Kβ) keV, likely an impurity of tin and silver at 22.16 (Kα) and 24.94 (Kβ) keV [45].

### 3.2. Main Ingredients: Semi-Quantitative View

**Figure 5:**
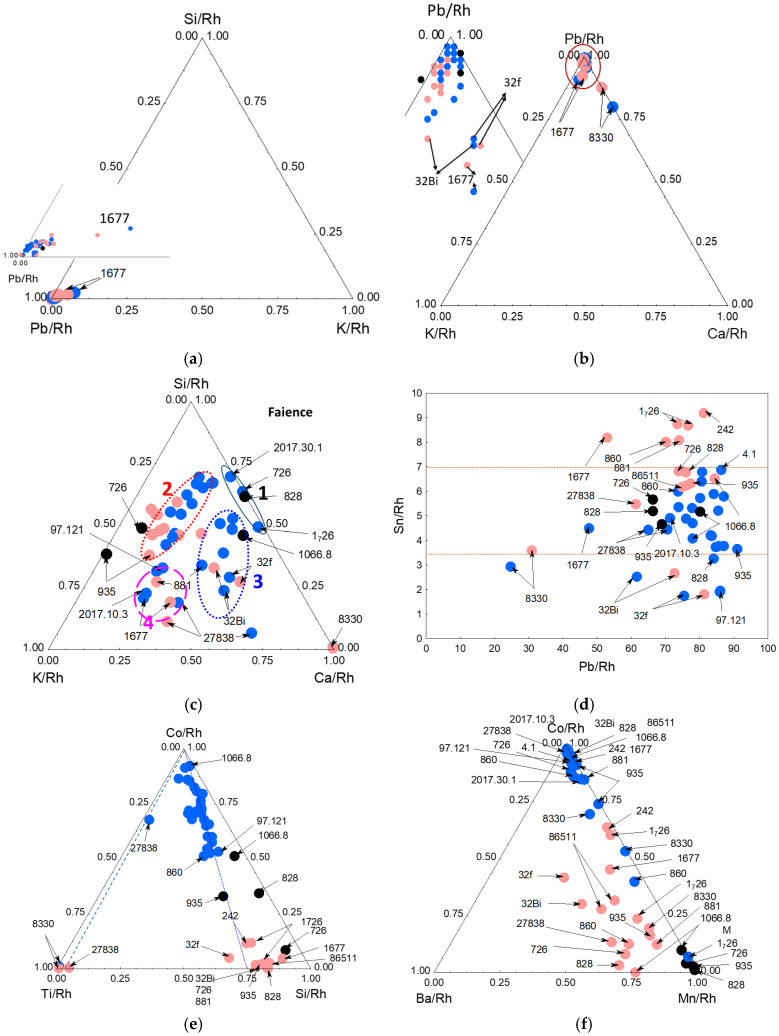
Si-Pb-K (**a**), Pb-K-Ca (**b**), Si-K-Ca (**c**), Co-Ti-Si (**e**), Co-Ba-Mn (**f**) ternary and Sn-Pb binary (**d**) diagrams constructed from the corresponding element peak areas of blue and white glaze. Only the inventory number without mention of the beneficiary museum (Nevers: NF; Sèvres: MNC) is indicated. The colors of the circles roughly match those of the decor: salmon: white; blue and black. Lines and ellipses are guides for eyes.

Figure 5 confirms that all the glazes are rich in lead but that two stand out, one by a slightly richer potassium content (the “Water bearer” statuette NF 1677) and the other by a higher calcium content (the “Workshop sign” MNC 8330). The Pb-Ca-K ternary diagram confirms the particularity of NF 1677 and MNC 8330 glazes but also points out the special composition of the two oldest artifacts, the tiles of the ducal palace (NF 32). The ternary diagram of the relative areas of the peaks of the elements Si, K and Ca shows the variability of the compositions of the glazes. In some cases the composition of the white glaze is close to that of the blue one; in other cases the difference is obvious. We can distinguish different groups: the first one named “1” is almost free of potassium (2017.30.1, 726, 828 and 1_7_26 that correspond only to blue areas); two groups named “2” and “4” which are characterized by a comparable level of calcium and the group “3” whose K/Ca ratio is almost constant but with more or less silica. We can think that these groups correspond to common workshops or periods due to the use of different recipes or raw materials. These productions being artisanal, the distribution of data is wide, much wider than what we observe for glazed porcelain from factories, for example Sèvres [18] or Meissen [19]. The fact that we observe that certain groups are specific to the blue color demonstrates that the material providing the cobalt is of a different composition from the glaze: group 1 is almost potassium-free, which excludes the use of smalt as a cobalt source and instead suggests the use of saffre (a cobalt-rich slag, with or without added silica, produced during the extraction of silver, bismuth, or arsenic) or cobalt compounds (e.g., sulfate) [18,19,27]; group 4 is richer in potassium, consistent with the use of smalt; group 3 consists almost exclusively of data from blue areas, which is consistent with the use of a cobalt source that also contains calcium.

**Figure 6:**
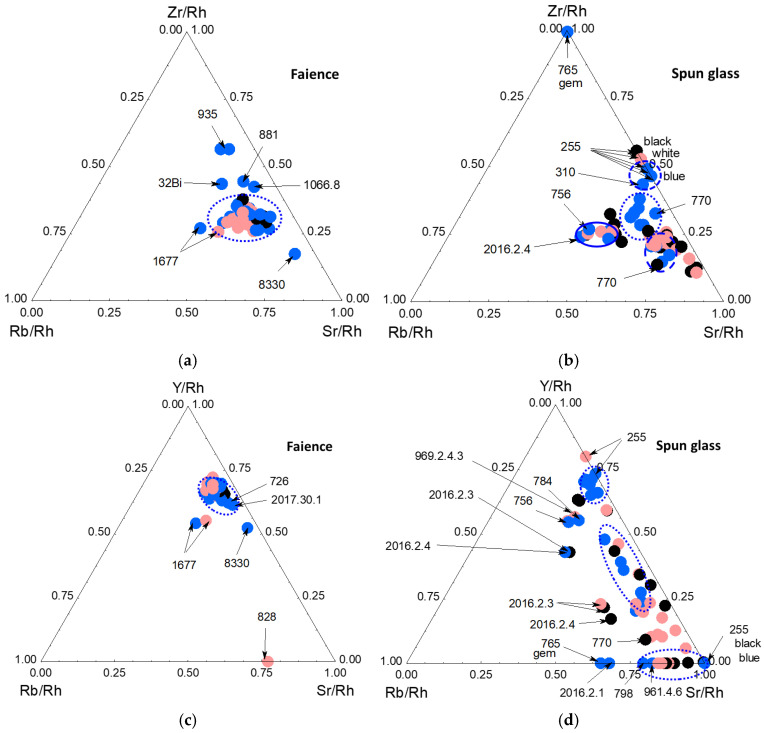
Left, Zr-Rb-Sr (**a**) and Y-Rb-Zr-Sr (**c**) ternary diagrams constructed from the corresponding element peak areas of blue and white faience areas. Comparison with data obtained from spun-glass figurines assigned to the Nevers city workshop (right, (**b**,**d**), after data of [70]; for more information on the spun-glass figurine, see the reference).

It is well established that the XRF signal of certain impurities is intense enough to be used to identify common usage of the same raw materials. This is the case for the zirconium (Kα peak at 15.78 keV) and yttrium (Kα peak at 14.96 keV) elements, impurities mainly of quartz in the form of zircon inclusions [64,65,66]. However, the yttrium Kα peak is located at the same energy as a lead peak. Strontium (Kα: 14.17 keV) (impurity of minerals containing calcium) and rubidium (Kα: 13.40 keV) (impurity of minerals containing sodium and/or potassium) are also well visible on spectra [67,68,69]. Figure 6 compares the ternary diagrams of these impurities obtained for the earthenware studied here and spun glass produced at the same time in Nevers [70]. There are similarities and differences, with most of the glass used to make spun-glass figurines probably being imported. For the faience glazes, we observed a very low dispersion of the data, particularly for the white glazes, which supports the use of the same recipe and raw materials for all white-opacified glazes. The heterogeneity is stronger for the blue, which indicates the use of several sources of cobalt. Apart from the potter’s sign plate MNC 8330 (dated 1658), we find the NF 935 (plate dated 1810) and, to a lesser extent, NF 881 (ewer, 18th c.), NF 1066.8 (stout, 18th c.) and NF 32B (initial, 1588–1589). These cobalt compositions outside the central group correspond to the oldest pieces and the most recent objects.

The Co-Ti-Si and Co-Ba-Mn diagrams show that the Ti/Si ratio distinguishes NF 27838 and MNC 8330 Ti-rich glaze, although NF 828, NF 1066.8, and NF 726 black areas are Ti-poor.

The concentration measurements show great unity in the use of the same raw materials providing silica and fluxes over a large period, contrary to what is observed for spun-glass figurines, according to the use of different imported glass trays for the latter [70]. As a preliminary conclusion, most of the cobalt used for earthenware corresponds to one of the types of cobalt used for spun glass.

### 3.3. Comparison After z-Score Normalization (Glassy Matrix)

**Figure 7:**
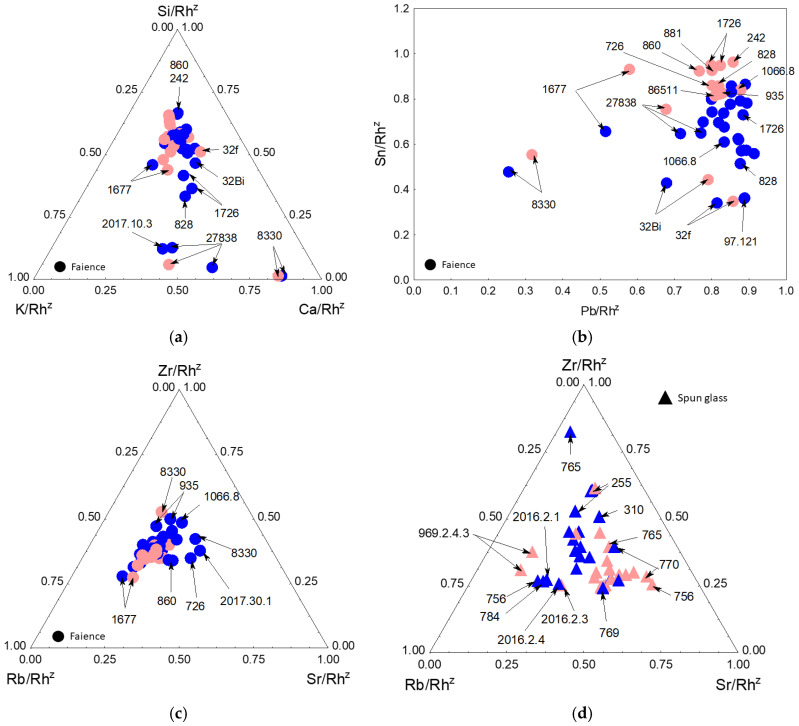

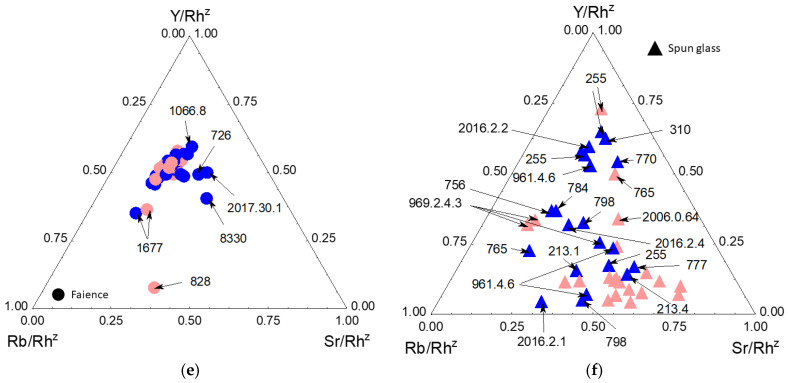
Si-K-Ca (**a**), Sn-Pb (**b**), Zr-Rb-Sr (**c**), and Y-Rb-Sr (**e**) diagrams constructed from the corresponding element peak areas of the blue and white areas of faience (solid circles) after z-score standardization. Comparison with data obtained from spun-glass figurines (solid triangle, (**d**,**f**)) assigned to the Nevers city workshop (right, after data of [58]; for more information on the spun-glass figurine, see the reference [70].

Figure 7 compares the ternary diagrams related to sand impurities (Zr and Y) and flux impurities (Rb, Sr), identical to those presented in Figure 5, but after z-score standardization in order to take into account the intensity variations in each characteristic peak, whose intensity primarily depends on the element considered. This standardization has little effect on the clustering: for the faience objects, most of the data form a much more compact group, far better defined than for the spun-glass figurines. The objects that do not belong to the main group are generally the same. For the Si–K–Ca ternary diagram corresponding to the major elements, the objects MNC 83330, MNC 27838, 2017.10.3, NF 828, and NF 1726 are clearly differentiated. The NF 32 tiles lie at the periphery of the group, but they are clearly distinguished in the Sn vs. Pb biplot. Similar conclusions can be drawn when considering the ternary diagrams related to impurities. The very low variability of the faience glazes is evident when compared with the equivalent diagrams for the spun-glass figurines [58]. In contrast to the spun-glass objects, for which the data dispersion is large, the faience data form a rather well-defined cluster with only a few outliers. No major differences are observed between the blue and white areas of the faience objects, except for the lower Rb content in the blue-colored areas.

### 3.4. Cobalt and Associated Elements: Semi-Quantitative View

**Figure 8:**
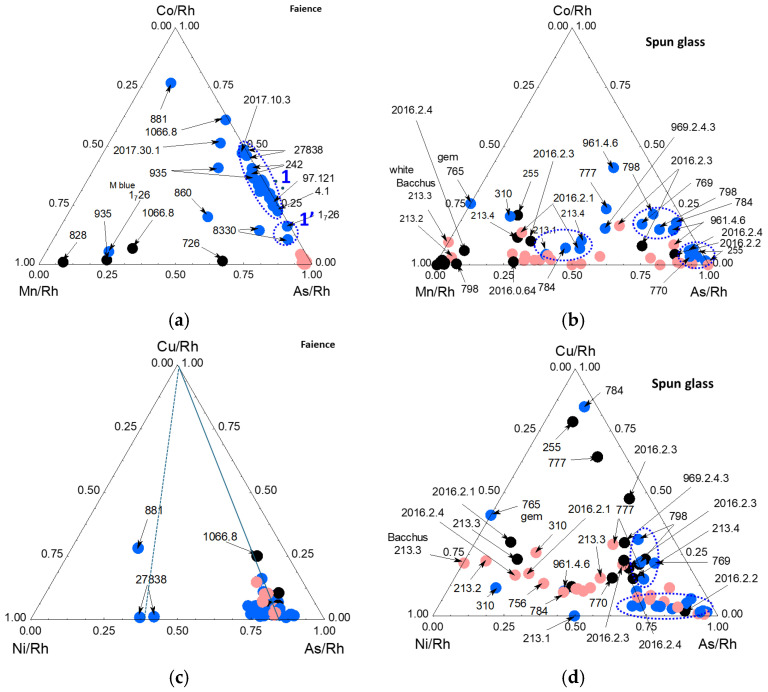

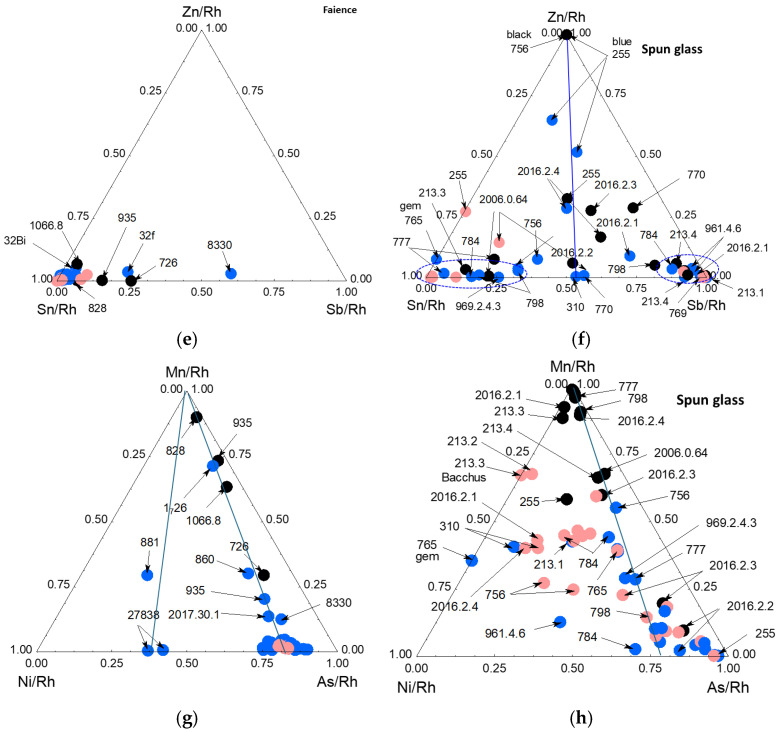
Co-Mn-As (**a**,**b**), Cu-Ni-As (**c**,**d**), Zn-Fe-Ti (**e**,**f**) and Mn-Ni-As (**g**,**h**) (all normalized to Rh signal) ternary diagrams constructed from the corresponding element peak areas of blue and white glaze ((**a**,**c**,**e**,**g**) “faience”). Comparison with data obtained from spun-glass figurines ((**b**,**d**,**f**,**h**), “spun glass”, right column) assigned to the Nevers city workshop (right, after data of [70]).

**Figure 9:**
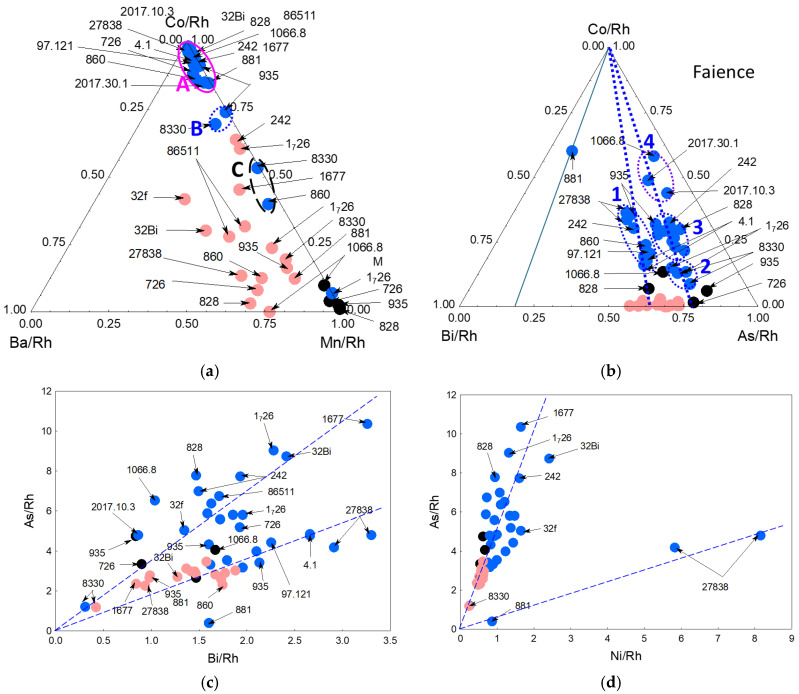
Co-Ba-Mn (**a**), Co-Bi-As (**b**), As vs. Bi (**c**) and As vs. Ni (**d**) (all normalized to Rh signal) diagrams constructed from the corresponding element peak areas of blue and white glaze. The groups are called 1, 2, etc. or A, B, etc.

Figure 8 and Figure 9 compare the areas of the peaks relating to the elements potentially associated with cobalt (As, Mn, Ni, Zn, Fe, Bi and Cu). In Figure 8, a comparison is also made with the Nevers spun-glass data [70].

The variation in cobalt content along a line toward the Co triangle summit reflects the degree of blue coloration: higher cobalt concentrations are required for darker blue hues. All data located on a line correspond to blue décor made using the same material providing cobalt element. The intersection of the line with the triangle side opposite the cobalt summit gives the common elemental ratio. The Co-Mn-As diagram in Figure 7 confirms that, unlike spun glass, there is a main group 1 (with perhaps two subgroups) and special cases, most of them already identified from the impurities (MNC 8330, NF 935 (plate, 1810), NF 881, NF 1066.8). The Cu-Ni-As and Mn-Ni-As diagram clusters are well separated with different objects decorated with cobalt richer in nickel (ewer NF 881 and large dish NF 27838) or with less arsenic (NF 881 and NF 1066.1, 2017.3.1, 935 and 860). The Co-Ba-Mn and Co-Bi-As diagrams in Figure 9 confirm these differences and the Bi/As ratio makes it possible to differentiate the main group into two subgroups, one a little richer in bismuth. The fact that the barium signals are higher for white glazes than for blue ones and that the silicon signals are different (Figure 5) indicates that cobalt is provided in most cases by a glass rich in cobalt but free of barium. We know that cobalt is a by-product of the exploitation first of silver, bismuth and arsenic and secondarily of copper. Mines operated solely for cobalt are exceptional in Europe. The common assumption of considering the Erzgebirge (Johanngeorgenstadt, Annaberg, Schneeberg, Marienberg, Freiberg and Joachimsthal) [22,27] as the single source of cobalt for blue decoration of European pottery should probably be reviewed, and efforts must be made to find information in the archives relative to other European mining places, such as Schwartzwald, Thuringia and Harz in Germany; Sainte-Marie-aux-Mines in France [22,27]; Giftain in Catalonia [18,19,22,27], etc., most of them having been active since almost the Middle Ages. The Schneeberg mine, exploited since the early Middle Ages, is known to also produce bismuth [71]. On the other hand, the neighboring Freiberg mine also produced silver and cobalt but with zinc and uranium [72,73,74]. Thus, these mines may represent potential sources of different cobalt ores; however, other sources cannot be excluded, as current knowledge of the characteristics of each mining area remains insufficient for reliable attribution. Mn/As, Ni/As, Bi/As, and Sn/Sb are effective criteria for distinguishing the different types of cobalt used. The degree of purification also plays a role in determining the final purity of the cobalt source. The As vs. Bi and As vs. Ni biplots confirm that different types of cobalt are used.

### 3.5. Comparison After z-Score Normalization (Blue Areas)

**Figure 10:**
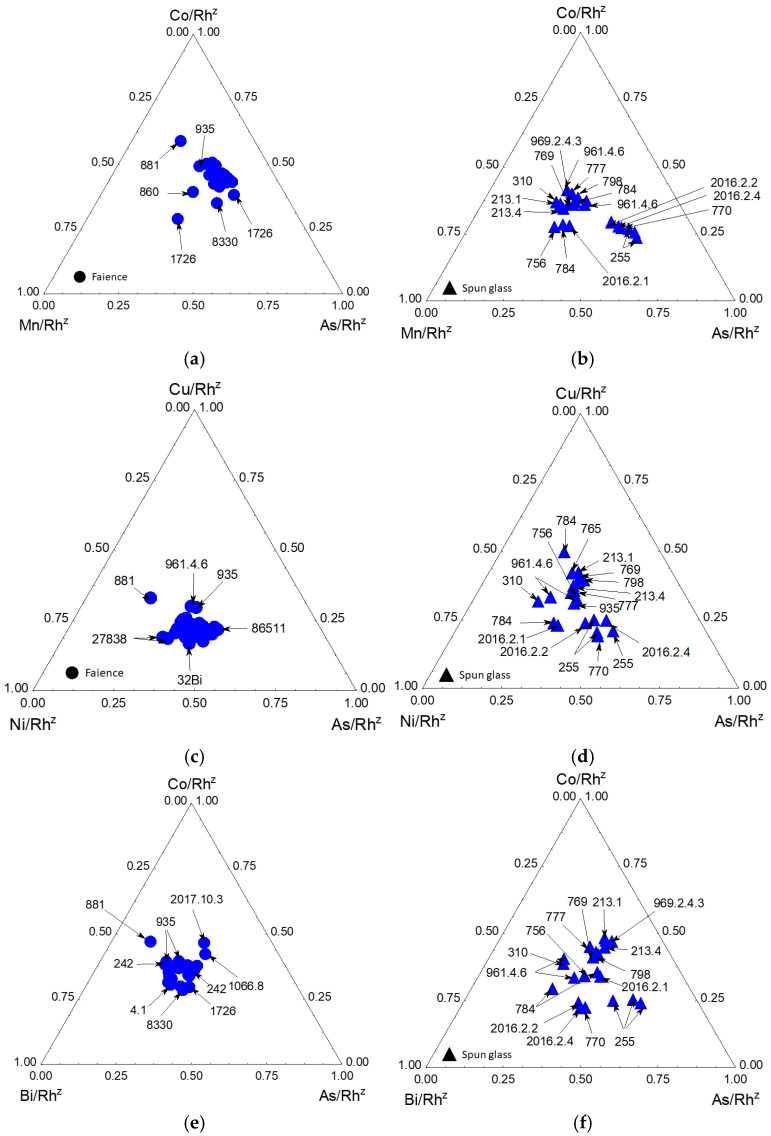
Co-Mn-As (**a**,**b**), Cu-Ni-As (**c**,**d**) and Co-Bi-As (**e**,**f**) ternary diagrams constructed from the peak areas of the corresponding elements measured in blue and white areas of faience ((**a**,**c**,**e**), solid circle) after z-score standardization. Comparison with data obtained from spun-glass figurines ((**b**,**d**,**f**), solid triangle) attributed to Nevers city workshops (right, data from [70]; for further information on the spun-glass figurines, see the corresponding reference).

Figure 10 compares the diagrams presented in Figure 9 using data standardized by the z-score method for the faience objects and the spun-glass figurines, focusing on cobalt and the elements geologically associated with cobalt. In these diagrams, several types of cobalt are clearly evident for the spun-glass objects, whereas the faience objects form a central cluster together with a few objects whose cobalt composition differs from the main group. The standardization enhances the definition of the clusters by eliminating variability resulting from differences in precision between the elements. The objects differing from the central group are again identified, namely the statue of Sta Magdalena NF1726, the workshop sign MNC 83330, two early nineteenth-century objects, NF 860 and NF 881, as well as the decorated plaque 2017.10.3 and NF 1066.8 stout dated to 1845. However, the differences between measurements performed in areas of the same color within a single object illustrate the intrinsic variability, which is comparable to the diameter of the marked ellipses.

### 3.6. Black Color

**Figure 11:**
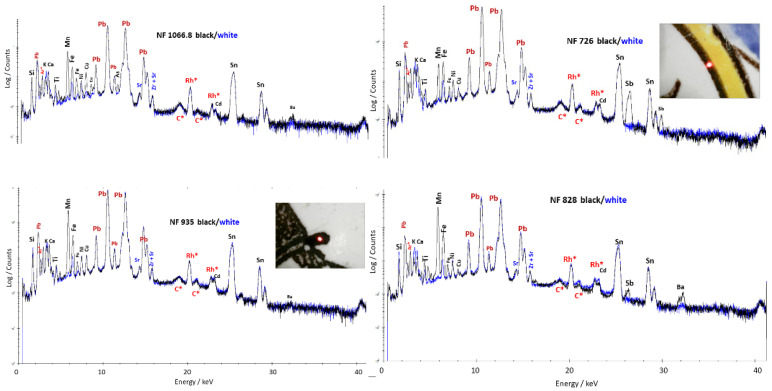
Comparison of XRF spectra recorded on black areas for century objects (see Table 1).

Figure 11 compares the spectra obtained in the dark areas. The significant increase in the manganese Kα peak at 5.9 keV is evident. The iron Kα peak at 6.4 keV also increases, but it contains the manganese Kβ component at 6.40 keV. The diagrams in Figure 7 and Figure 8, including the vertex Mn, confirm the use of this element for blacks, the black signal.

## 4. Discussion

The impurity signatures of the sands and fluxes indicate that, except for a few objects, all glazes, whether from the early production phase or after 1800, were made using the same raw materials for the vitreous matrix. This is totally different from the spun-glass figurines produced during the same periods in the city of Nevers due to the importation of glass rods of different compositions and origins [70]. A wide dispersion of flux and tin contents is observed (Figure 5). However, given the significant depth of measurement for the tin peaks located around 25 and 29 keV (see, e.g., Figure 9), the variations observed may result from the variation in the thickness of the glaze layer opacified with cassiterite, which causes a variable contribution to the XRF signal from the underlying paste, paste free of tin. Three objects, NF 935 (plate dated 1810 representing the Syndic de marine Dupuis), NF 1677 (the famous water bearer with candle decoration dated stylistically from 1660 to 1680) and MNC 8330 (potter’s sign plaque bearing the date 1658), are made using a glaze obtained from different raw materials. The multiplicity of polychromy of the decor of the later artifact can explain a special manufacturing by a master potter. The candle decoration is characteristic of a period of Nevers production, and it would be appropriate to analyze around ten pieces of this type to see if the iconic piece analyzed in this work is representative of the entire series. The piece dedicated to the Syndic playing an important role in the “export” of Nivernais production corresponds to the post-revolutionary period of renewal of motifs and therefore perhaps also of the supply of new raw materials. Here too, to conclude, a much larger number of post-revolutionary objects must be studied.

### 4.1. Classification Using PCA

**Figure 12:**
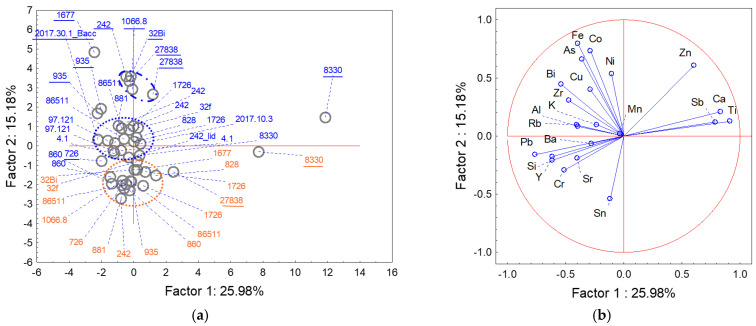

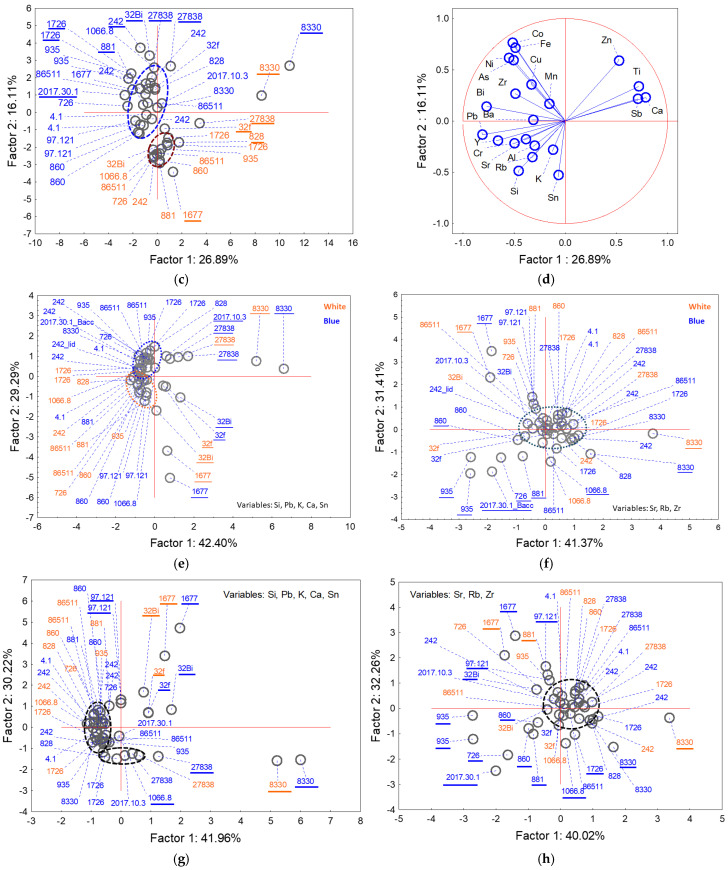
Comparison of PCA factor plots calculated using all elements measured with pXRF (**a**–**d**) and limited to two sets of variables, major elements (Si, Pb, K, Ca, and Sn, (**e**,**g**)) and the characteristic trace elements (Sr, Rb, and Zr, (**f**,**h**)). Elements that do not belong to the main group are underlined. Calculations with (**c**,**d**,**g**,**h**) and without (**a**,**b**,**e**,**f**) z-score standardization are shown. Inventory numbers of outliers are underlined.

**Figure 13:**
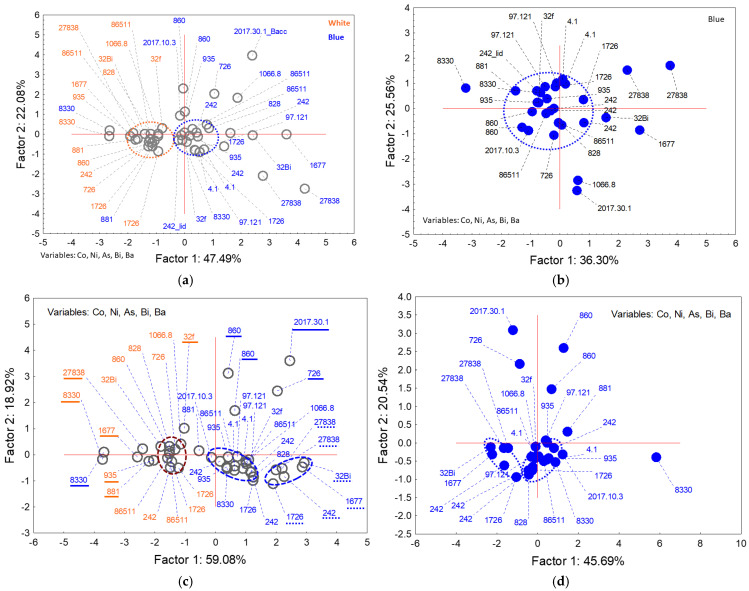
Comparison of PCA factor plots calculated using elements associated with cobalt for the white and blue glazed areas. Calculations with (**c**,**d**) and without (**a**,**b**) z-score standardization are shown.

Elemental composition, and in particular trace-element content, is widely used to identify raw materials, fluxes (Rb or Sr), and sands (Yn or Zr) [64,67,68,69]. Principal component analysis (Figure 12 and Figure 13) and hyperspace visualization of similarity distances (Figure 12 and Figure 13) have a long history in archaeometry since the 1970s [75,76,77,78,79,80]. These methods have primarily been applied to body paste analysis, due to the use of local raw materials (e.g., [78,79,80,81,82,83,84,85,86,87,88]), and have rarely been used for glaze elemental compositions [88]. In this study, they are applied to peak areas of characteristic elements showing clear signals in the XRF spectra.

Figure 12 compares the PCA factor plots calculated using the full dataset and reduced variable sets, namely major elements (Si, Pb, K, Ca, and Sn; bottom, left) and characteristic trace elements (Sr, Rb, and Zr) calculated from data normalized to the Rh source, without and with z-score standardization. The variance explained by the first two factors in the global model (ca. 26% and 15%) indicates a meaningful structuring of the data. The global PCA diagram confirms the results obtained through the ternary diagrams: only a few objects exhibit distinct enamel compositions, namely (from the most divergent) MNC 8330 (workshop sign), for which both white and blue glazes differ, and several objects with particular blue areas, including NF 1677 (Persian blue water bearer), NF 242 (large 17th c. blue-and-white plate), NF32 (16th c. tiles from the ducal palace), NF 27838 (large 18th c. blue-and-white plate), and NF 935 (1810 dated polychrome plate). The elemental loading diagram confirms that the MNC 8330 is distinguished by its high calcium content, as already indicated by the ternary diagrams in Figure 5, while group separation is mainly driven by cobalt and associated elements.

The PCA results calculated for both major and trace elements confirm all the observations previously obtained from the interpreted ternary peak-intensity diagrams and further show that objects NF 2017.10.3 and NF 2017.30.1 (two large decorative plates depicting Bacchus and a landscape ruin) also deviate from the main group. Note that the first two principal components explain approximately 40 and 30% of the variance, respectively.

Figure 13 presents the PCA obtained using only cobalt-related elemental data. The same objects that stand out from the main group are identified as in Figure 7.

Standardization with z-score markedly reduces data dispersion, and the clusters become better defined, characteristic of each color, white or blue, although the results remain very similar. With standardization, two clusters are more clearly observed for the cobalt-colored areas, together with several cases that do not belong to these clusters.

### 4.2. Classification Using Ward Similarity Diagrams

#### 4.2.1. Classification from Characteristic Impurities

**Figure 14:**
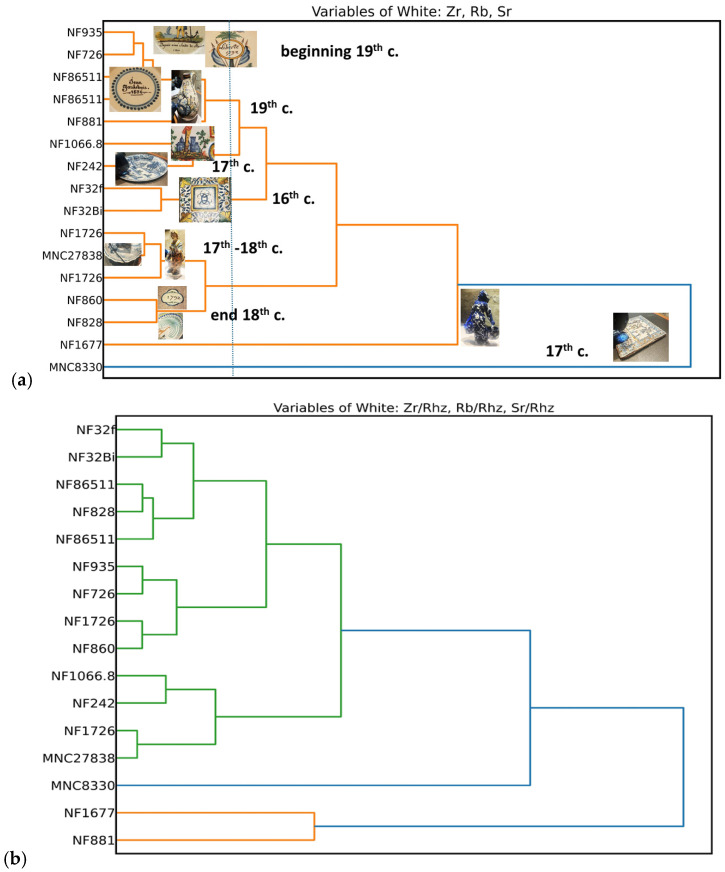
Ward Euclidean dendrogram of similarity built using Zr, Rb and Sr elements characteristic of white opacified glaze without (**a**) or after (**b**) z-score standardization.

The dendrogram constructed using the impurities whose peaks are clearly visible on the spectra, Zr, Rb and Sr (Figure 14), separates the objects into six groups; two objects are obviously apart: the potter’s sign plate (MNC 8330) and the statuette “The Water bearer” already clearly identified from the ternary diagrams.

The primary levels of classification are only weakly affected by standardization. It is clear that the glazes of objects NF881, NF1677, and MNC 8330 employed specific raw materials. The objects dating from the 19th century form a distinct group, consistent with the introduction of new raw materials. In contrast, the 17th century appears to involve the use of different raw materials.

#### 4.2.2. Classification from the Elements Associated with Cobalt

**Figure 15:**
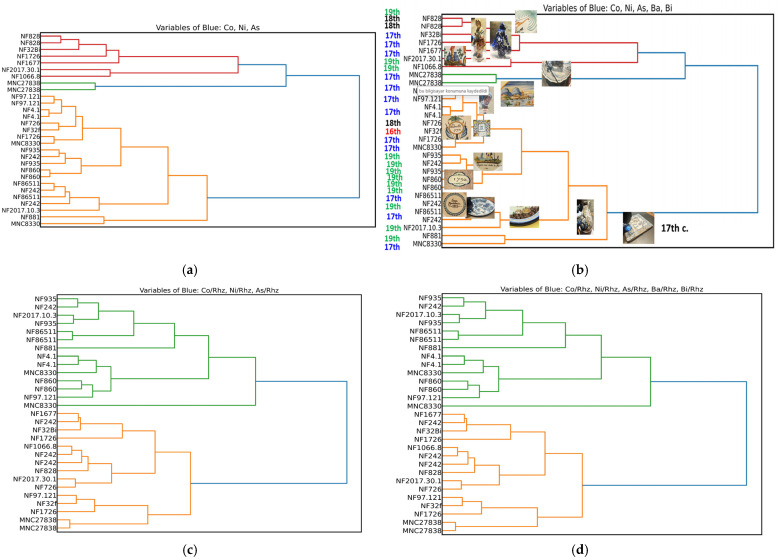
Ward Euclidean dendrograms of similarity built using Co, Ni, As (**a**,**c**), and Co, Ni, As, Ba, and Bi (**b**,**d**), elements characteristic of blue areas. Calculations with (**c**,**d**) and without (**a**,**b**) z-score standardization are shown.

Figure 15 presents the dendrogram constructed with cobalt and its associated elements. The dendrogram separates the corpus into two main groups (blue lines on the right) whatever the set of elements considered: Co, Ni, As, Ba, Ni or only Co, Ni and As. Most subgroups associate objects from the same century, although some intermixing is observed, indicating that only the first level of discrimination is meaningful. Two cobalt “grades” are thus used. Roughly, a separation can be noted between 19th and 17th century productions.

When examining the data, the presence of colors close to the spot (black line rich in manganese, zone of other colors) must be considered carefully because we see that, in fact, the contribution to the signal is much larger than the zone of a few mm^2^ of the laser spot since defining the measurement zone, all the more so since the perpendicularity to the surface is imperfect.

## 5. Conclusions

These preliminary pXRF analyses carried out on-site on major artifacts assigned to Nevers workshops and dated demonstrate both the potential and the limitations of on-site study of enameled objects. It is possible to identify enameled objects made using similar glaze compositions and the same raw materials based on the intense signatures of a limited number of impurities, which are representative of the geological context and therefore of the origin of the sands and fluxes. The non-invasive study of outstanding artifacts has the important limitation that information on the glaze stratigraphy and the glaze/body interaction is limited, contrary to the study of shards. The consideration of the relative signals of cobalt-associated elements is also highly effective in discriminating between objects produced using different cobalt sources or different grades of cobalt [31,89,90]. Eighteenth-century books such as that of Valmont de Bomare indicate that around ten grades of “smalt”, probably the most widely used cobalt-bearing ingredient from the 16th to the 18th century, were available [91], undoubtedly at varying prices. While it may be hypothesized that higher-quality grades were reserved for more sophisticated objects intended for specific clients, this remains to be demonstrated through a study of a representative corpus. It appears evident that direct comparison in ternary diagrams of peak areas characteristic of elements linked to geological origin is effective, and that the “blind” use of chemometric tools (PCA and Euclidean Hierarchical Classification) does not provide large additional insight. The contribution deals mainly with the visualization of the results.

The comparison between two types of objects produced in the same period in the same city is particularly effective for assessing variability. Indeed, comparison with measurements of spun glass colored blue produced at Nevers shows a much wider distribution of impurity contents, compositions, and cobalt types due to the constraints imposed by spun-glass technology, namely, the use of different glass rods with varying melting points, and therefore different compositions and probably different origins. In contrast, with the exception of the relative proportions of the fluxes K, Ca, and Pb, which show some variability, the data for the enamels are highly clustered, except for a few objects that appear to have been prepared according to different recipes (in another city or workshop?).

The water bearer, one of the most iconic artifacts assigned to Nevers, does not belong to the main cluster. This deserves the study of a large series of similar artifacts decorated with white candle splash on a blue background.

Three artifacts, first MNC 8330 (polychrome potter’s workshop sign), followed by NF 32 (Nevers palace polychrome tiles) and NF 1677 (blue-and-white water bearer), were produced using a glaze technology different from that of the other faience objects.

Different types of cobalt sources were identified. One group, characterized by the absence of potassium (2017.3.1, NF 726, NF 828, and NF 1726), is consistent with the use of saffre or cobalt compounds. Because most of these artifacts date to the late 18th century, the use of chemical cobalt sources (e.g., cobalt sulfate) is likely. In contrast, for the 17th-century statue NF 1726, the use of saffre is more probable.

The oldest artifacts (the 16th-century NF 32 tiles and the late 16th- or 17th-century NF 97.121 *istoriata* plate) are characterized by low Sn content and moderate potassium levels. Based on these parameters, it would be interesting to analyze a larger series of *istoriata* plates, since their attribution to Nevers, Lyon, or Italy remains highly debated.

NF 27831 (a blue-and-white plate dated to 1765) and NF 881 (a polychrome ewer dated to 1805) were produced using a nickel-richer cobalt source. Although the diversity of cobalt sources used by potters is broadly similar to that observed in spun-glass production, different cobalt types were clearly employed both across different periods and within the same period.

A larger corpus and an analysis of available documentation on Nivernais workshops are needed to investigate this further. This preliminary work highlights the value of this type of study for a better understanding of production, providing information not only on the evolution of recipes and raw material sources, but also on the technical consistency of production. These original data can then be compared with those derived from stylistic analysis of shapes and decorations.

## Figures and Tables

**Table 1:** Studied artifacts and their characteristics. The largest dimension is given.

View	Inventory Number Description (H or D) Period	Analyzed Color (XRF)	View	Inventory Number Description (H) Period	Analyzed Color (XRF)
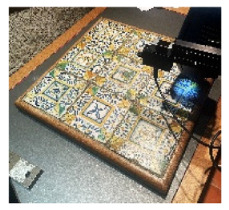	NF 32B (SC) C Initials (77.5 × 50 cm^2^) 1588–1589	White Blue Yellow Orange	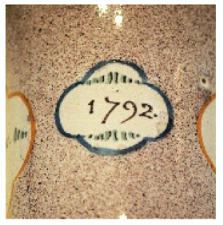	NF 860 Hanap (15.5 cm) 1792	White Blue Blue (black spot)
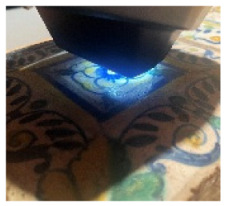	NF 32B (SC F) Flower (14.5 × 14.5 cm^2^) 1588–1589	White Blue Yellow	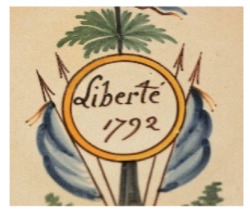	NF 726 Plate (22.5 cm) 1792	White Blue
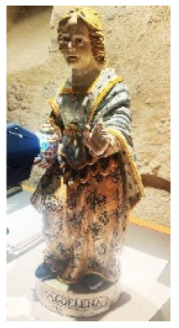	NF 1_7_26 Sta-Magdelena (66 cm) 1637	White Blue Yellow	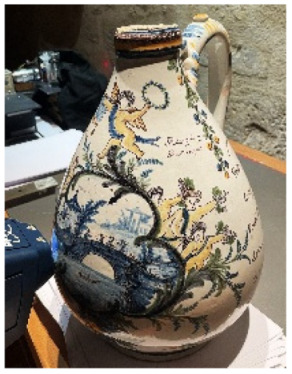	NF 881 Ewer (34.5 cm) 1805	White Blue
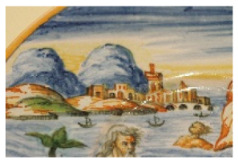	NF 97.121 Scylla et Glaucus “Gallatea con gliamour” mark, (D: 23 cm) c. 1640	Blue Yellow	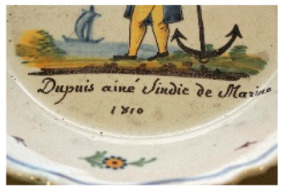	NF 935 Plate (22.5 cm) 1810	White Blue Yellow Orange Black
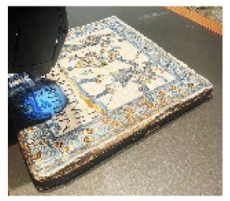	MNC 8330 Tile (35 × 35 cm^2^) 1658	White Blue	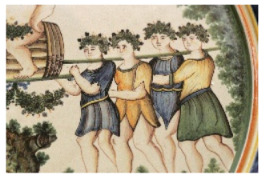	NF 2017.10.1 Decorate plate Depicting Bacchus (35 cm) 1820	Blue
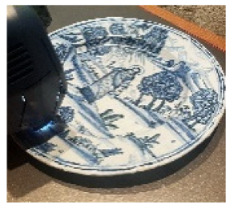	NF 242 Religious décor Dish (47.6 cm) 1665	White Blue Blue (dark)	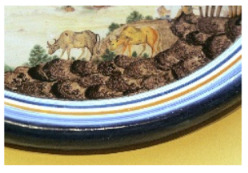	NF 2017.10.3 Decorate plate Depicting Ruins (33 cm) 1822	Blue
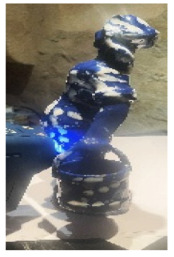	NF 1677 (62v9) candle décor (26 cm) ca. 1660–1680	White Blue	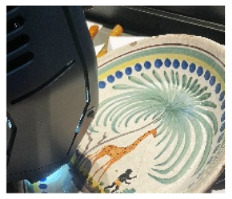	NF 828 Plate (22 cm) ca. 1828	White Blue Black
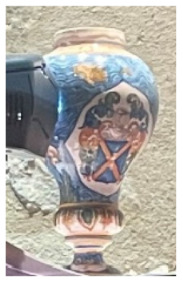	NF 4 Jar (35.5 cm) 17th c.	Blue Yellow	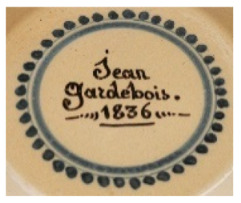	NF 965.1.1 Plate (20 cm) 1836	White Blue (dark) Blue
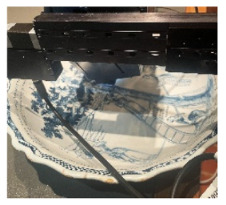	MNC 27838 Bowl ‘L’arbre d’amour’ (31.3 cm) 1765	White Blue	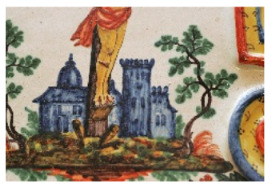	NF 1066 (Ben) Stout (46.8 cm) 1845	White Blue Black
			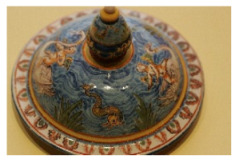	NF 4 modern lid (~5 cm) 1865	Blue

## Data Availability

The original contributions presented in this study are included in the article/Supplementary Material. Further inquiries can be directed to the corresponding author.

## Supplementary Materials

The following supporting information can be downloaded at: https://www.mdpi.com/article/10.3390/ma19122442/s1. Figure S1: XRF spectra of Nevers faience. Figure S2: Summary of the steps for processing XRF data files with Bruker Spectra Artax software (version 7.4.0.0); Table S1: XRF peak area after z-score standardization.
