# Supplementary material for: Nevers City Earthenware Blue Glaze: pXRF Categorization from Cobalt Sources and Raw Materials Impurities: Comparison of Reasoned and Chemometrics Methods
Source: Materials (Basel). 2026 Jun 7;19(12):2442. doi: 10.3390/ma19122442 (PMC13301694; doi:10.3390/ma19122442)

Nevers city earthenware blue glaze: pXRF categorization from cobalt sources and raw materials impurities. Comparison of reasoned and chemometrics methods.

Gulsu Simsek-Franci[1], Philippe Colomban[2*], Marie-Lys Chevalier[3]

[1] Yildiz Technical University, Faculty of Chemical and Metallurgical Engineering, Department of Metallurgical and Materials Engineering, Davutpasa Mah. Davutpasa Caddesi 34220 Esenler - Istanbul, Türkiye,

gulsu.simsek@yildiz.edu.tr

[2] Laboratoire 'De la Molécule au Nano-objet : Réactivité, Interaction et Spectroscopies, (MONARIS UMR8233), Sorbonne Université, CNRS, Campus P.-et-M. Curie, 4 Place Jussieu, 75005 Paris, France ;

philippe.colomban@sorbonne-universite.fr

[3] Musée de la faïence et des Beaux-Arts- 'Frédéric Blandin', 16 rue Saint-Genest, Nevers, France

*Correspondence: philippe.colomban@sorbonne-universite.fr

Figure S1

XRF Spectra – Nevers Faiences

(for each artifact, inventory number, description and expected date of production are given)

Inv. NF 32B, Tile (SC), 16th century (1588/1589)

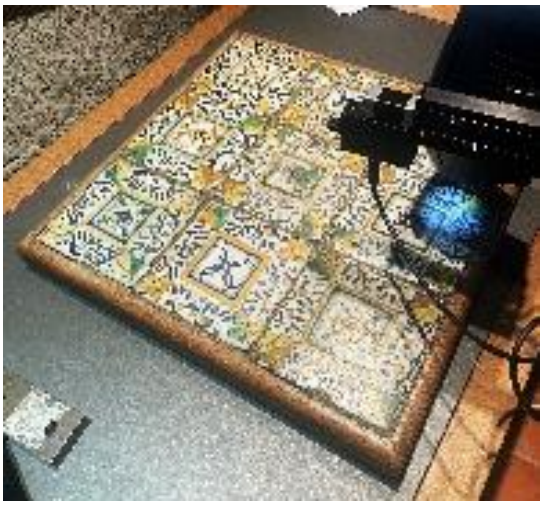

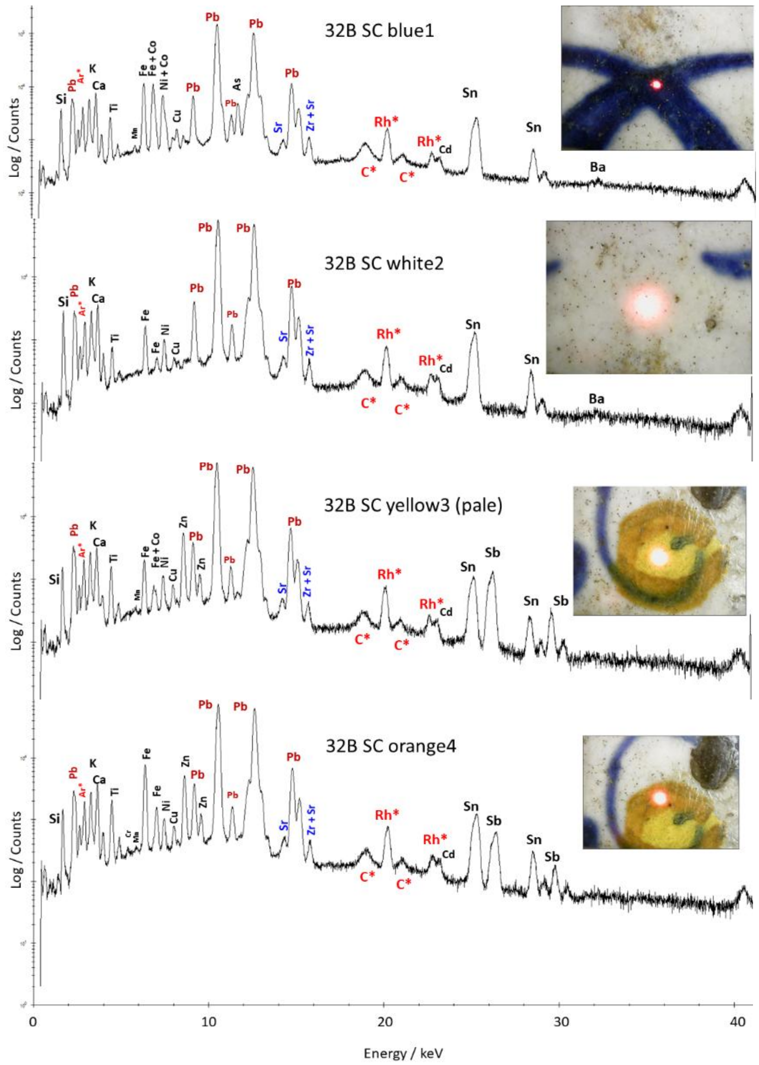

Inv. NF 32B, Tile (flower), 16th century (1588/1589)

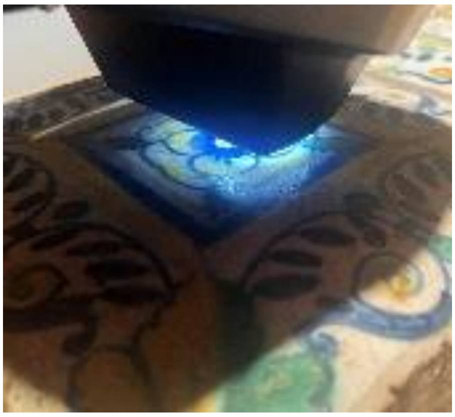

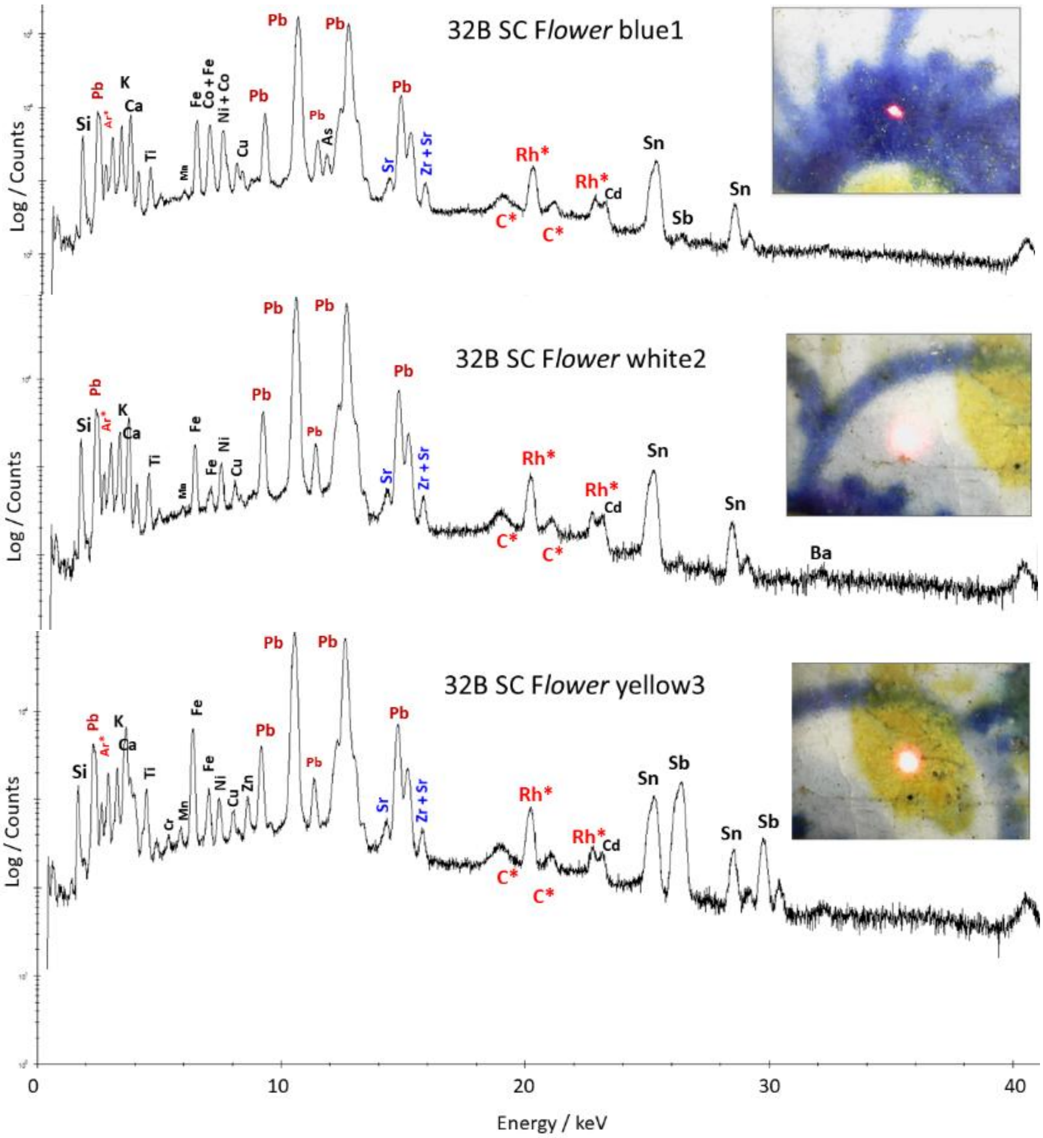

Inv. NF 1$_7$26, Ste-Magdelena (h: 66 cm), 1637

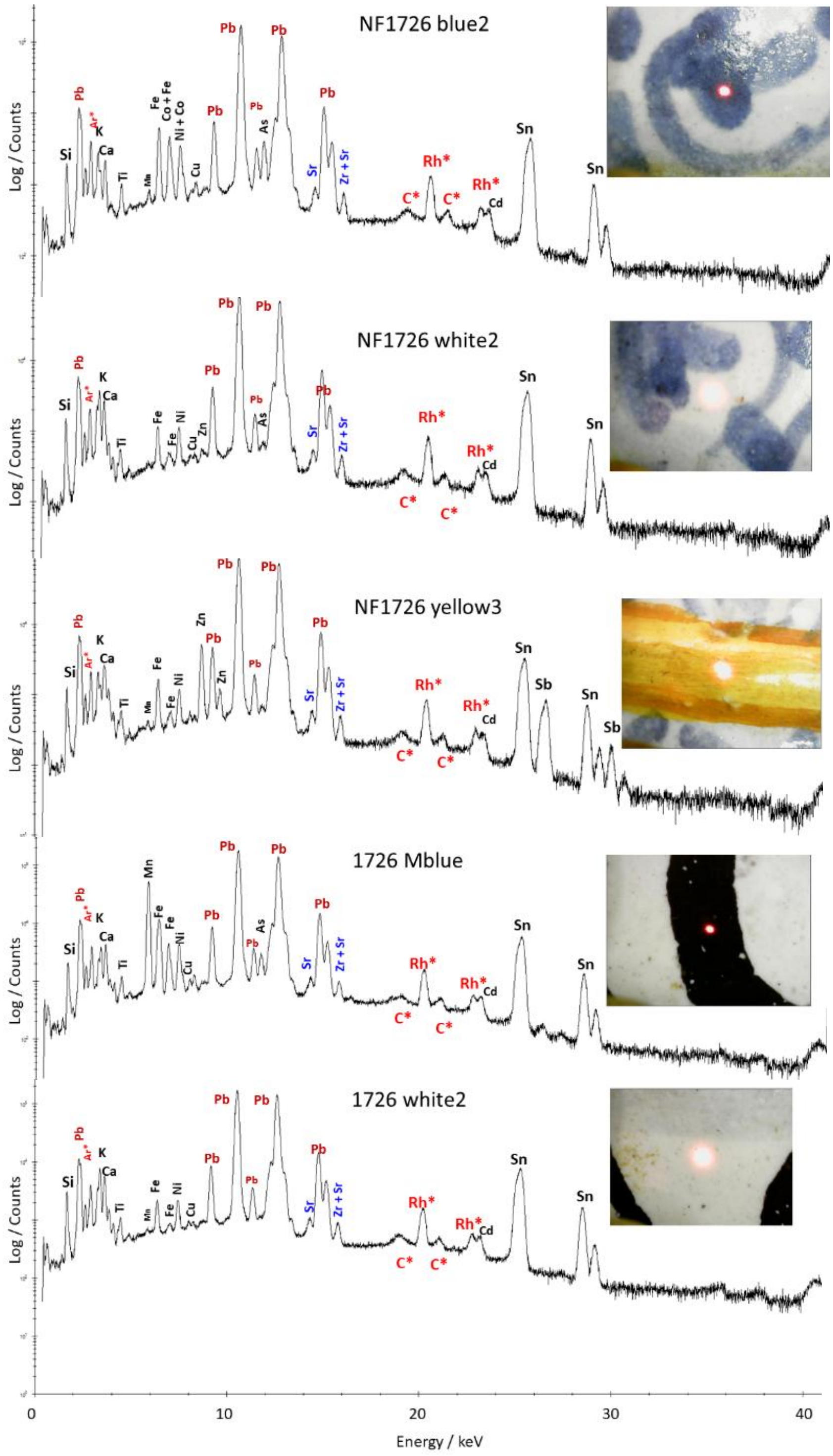

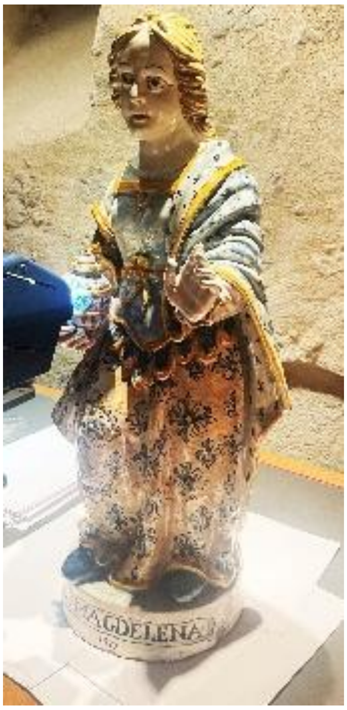

Inv. NF 97.121, Scylla et Glaucus (“*Gallatea con gliamour*” mark) plate, ca. 1640

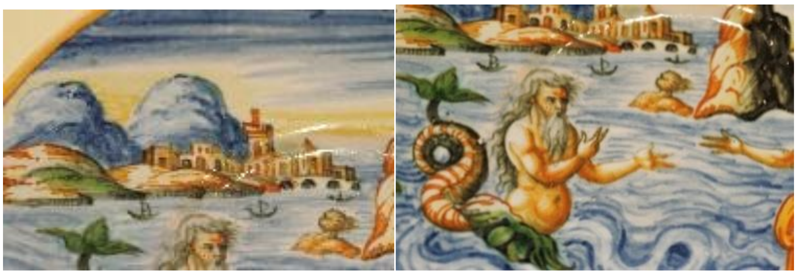

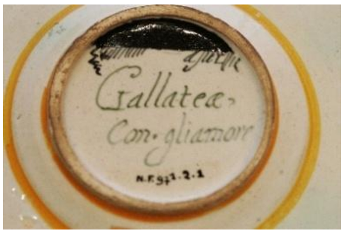

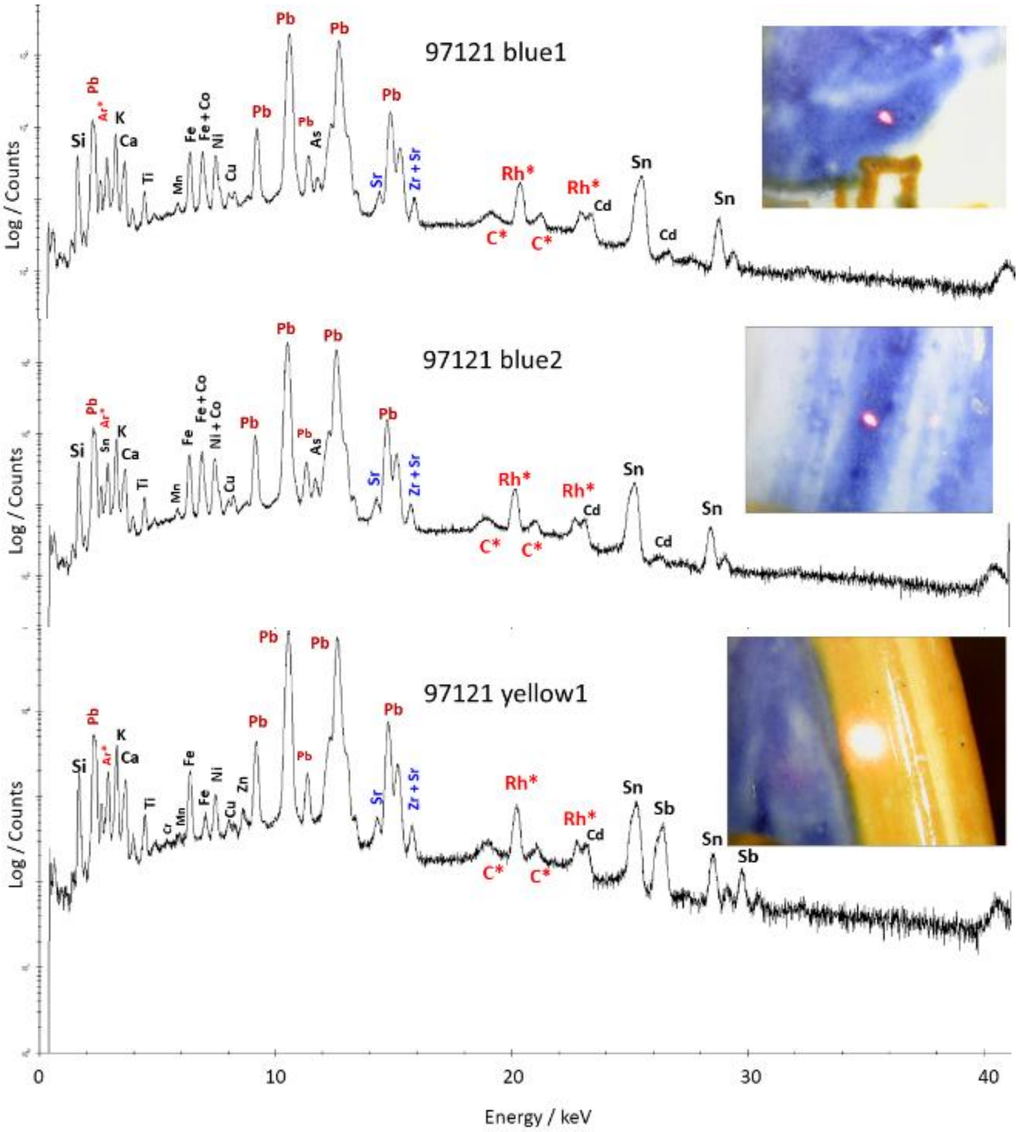

Inv. MNC 8330, Tile, 1658

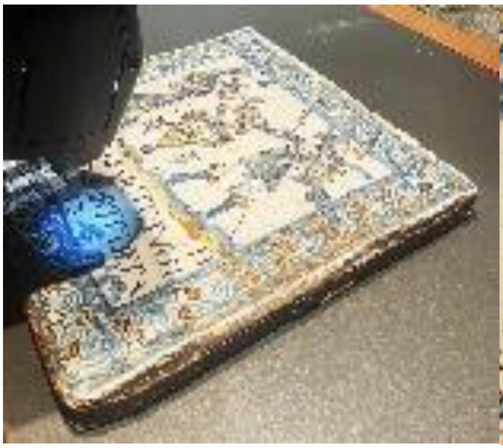

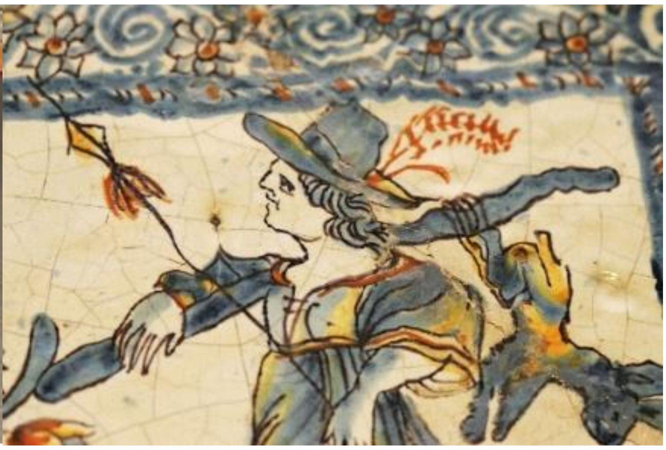

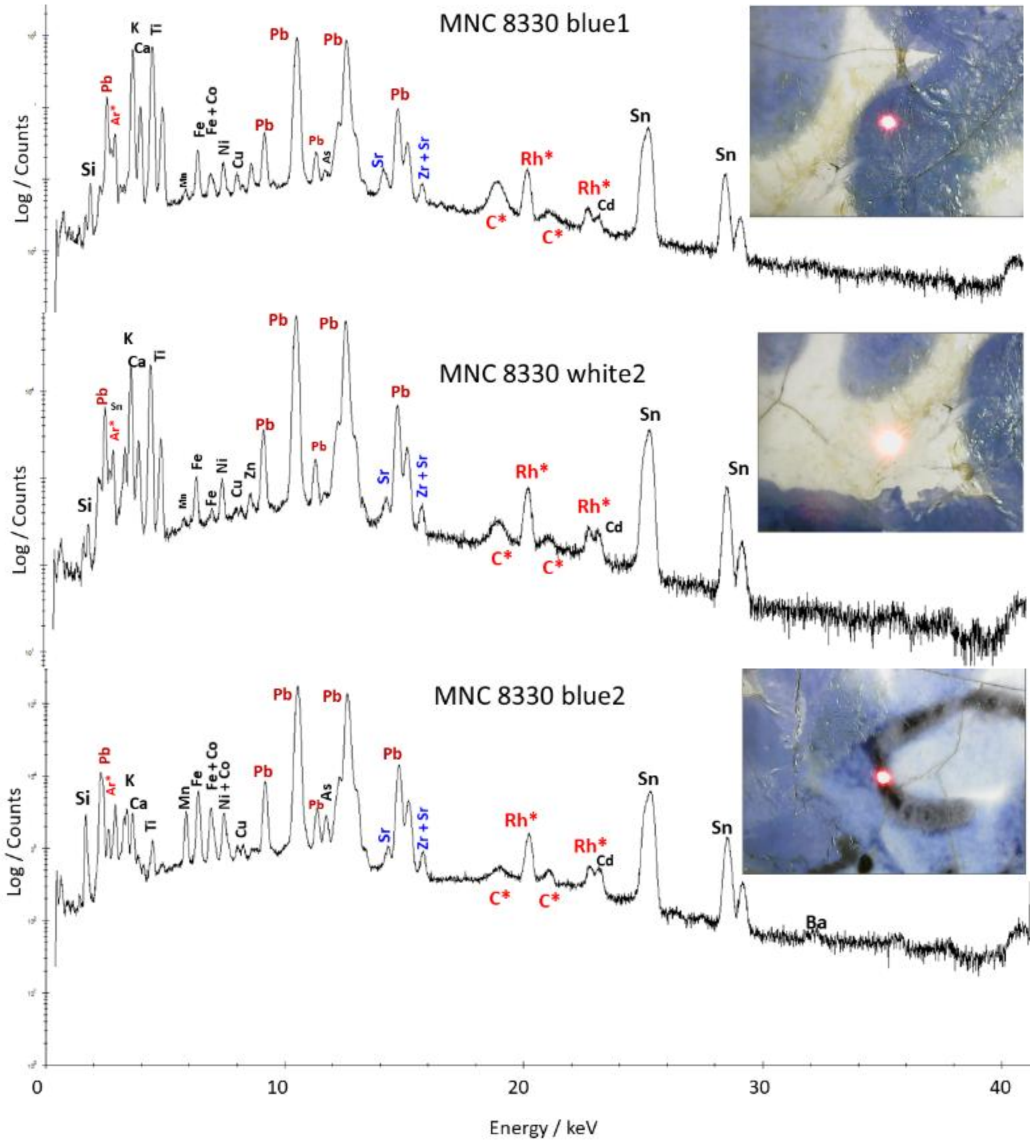

Inv. NF 242, Religious decor dish (d: 47.6 cm), 1665

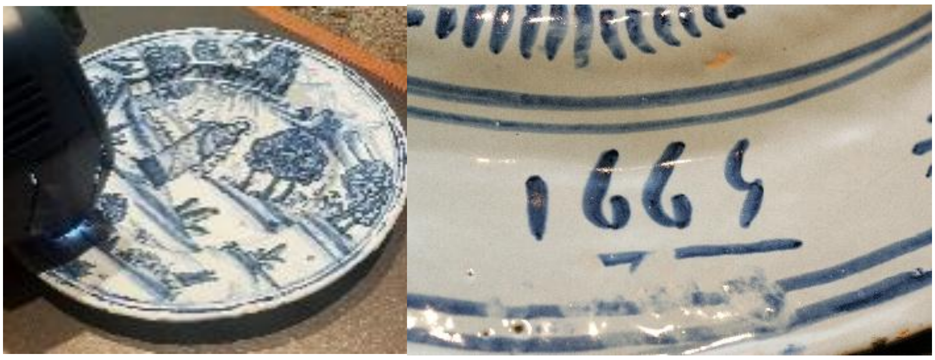

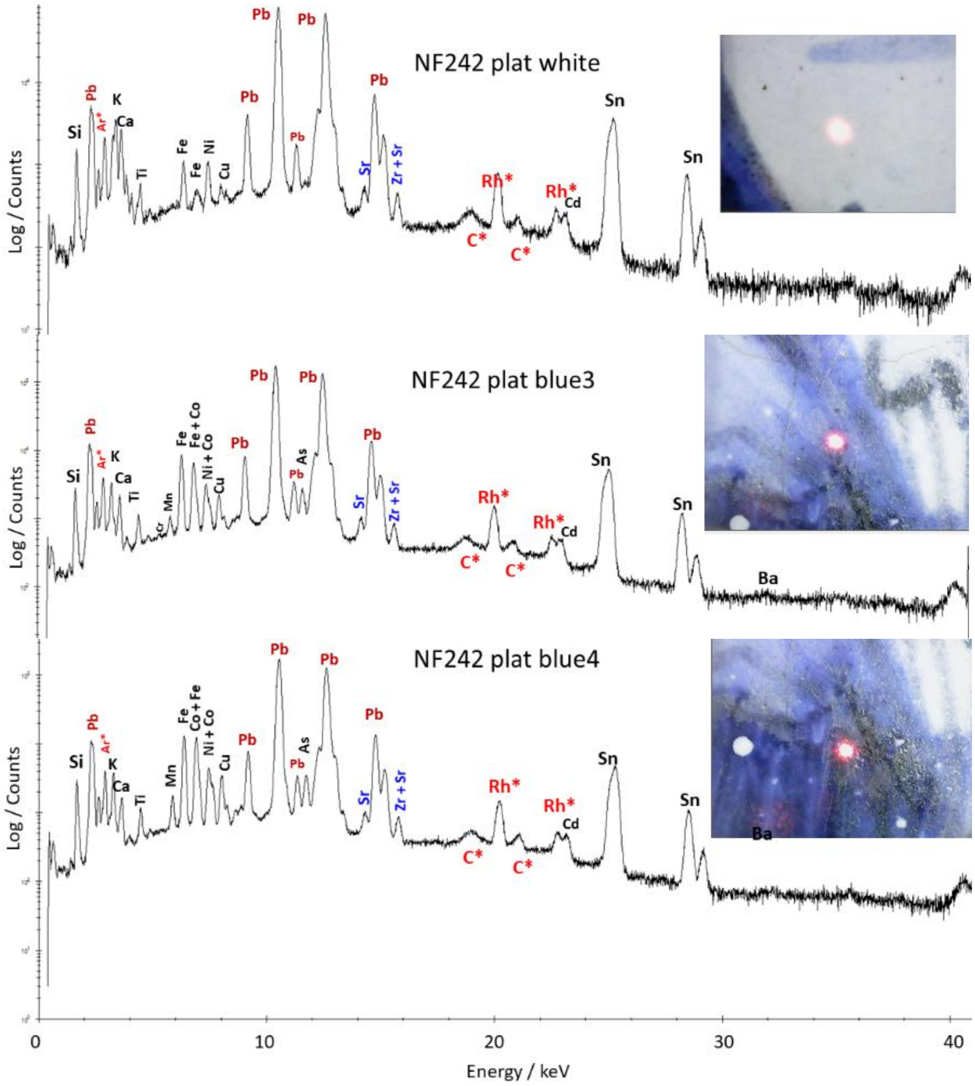

Inv. NF 4.1, vase, 17th century

Inv. NF 1677, candle spots water bearer (h: 26 cm), ca. 1660-1680

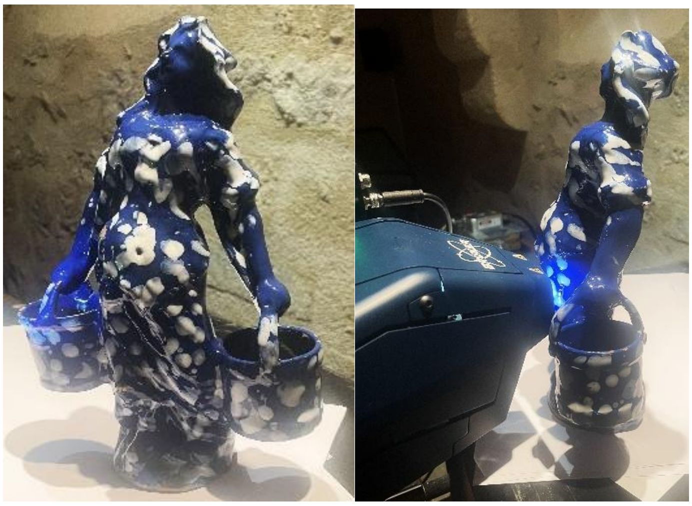

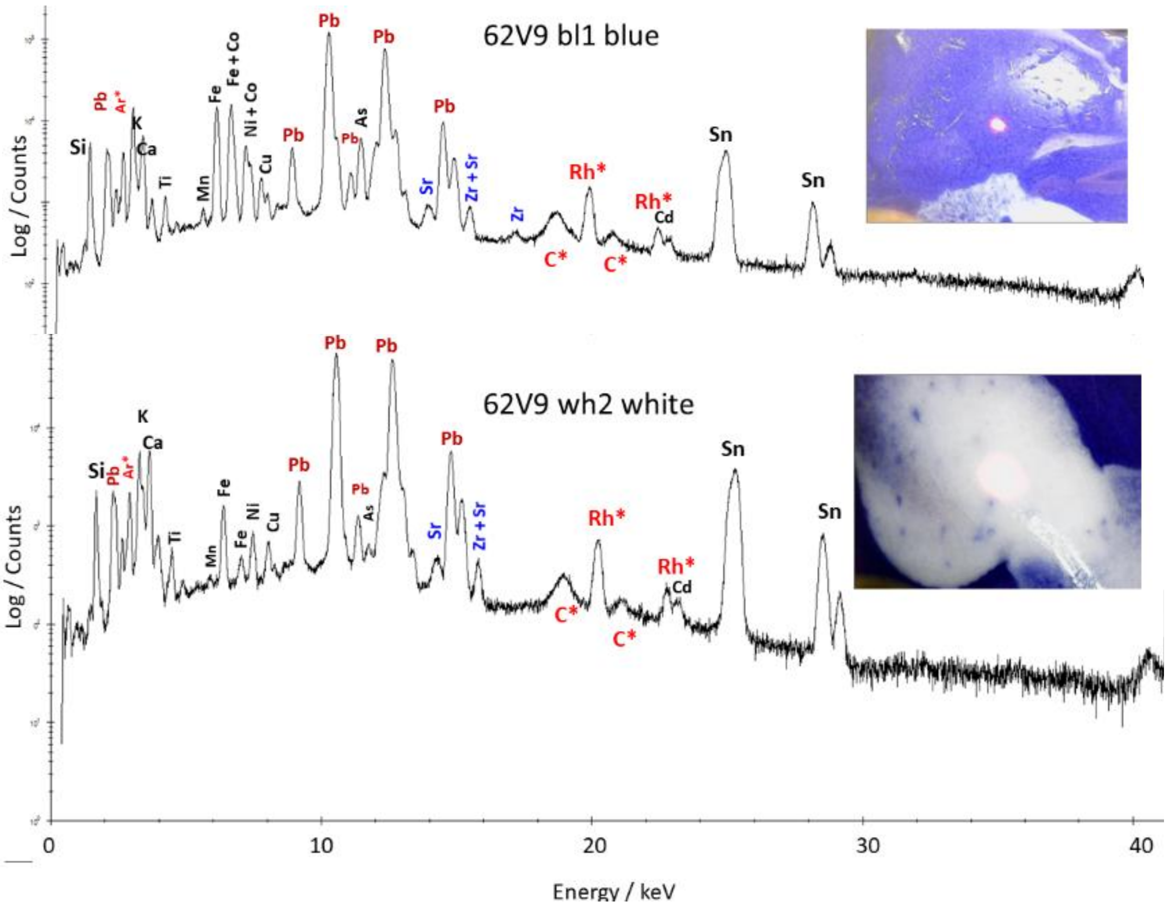

Inv. NF4, Jar (h : 36.5 cm), 17th century

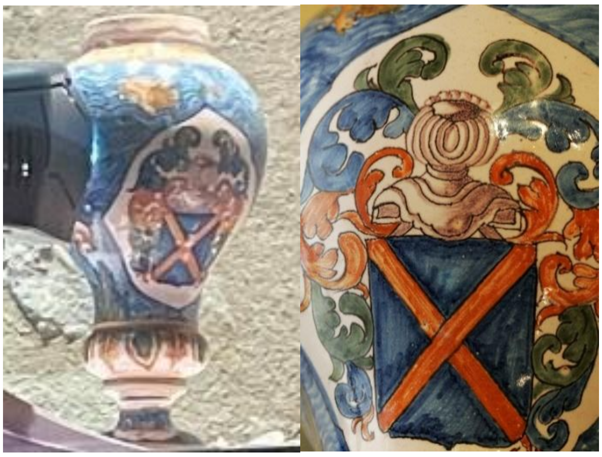

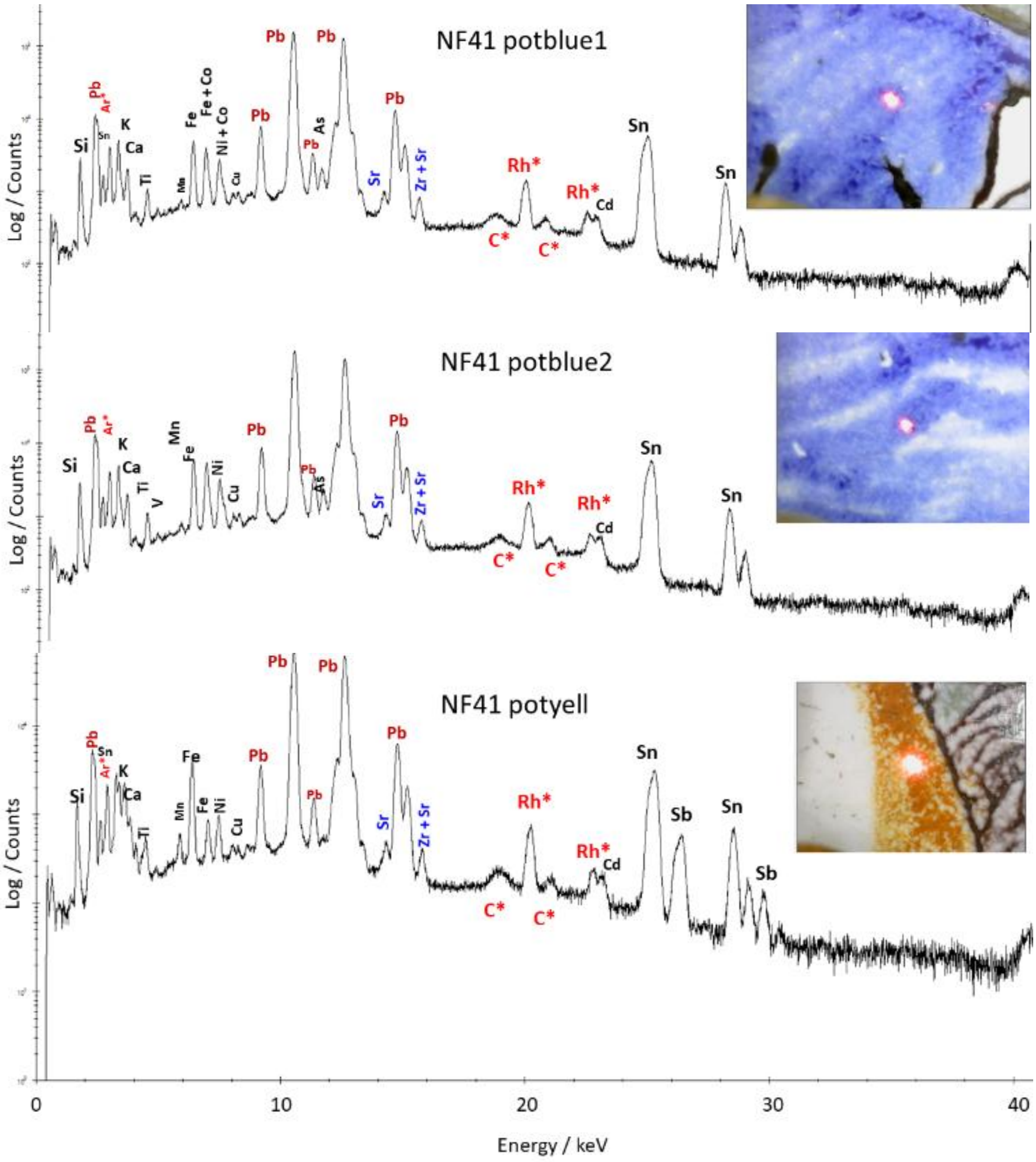

Inv. NF 4.1, lid, 1865

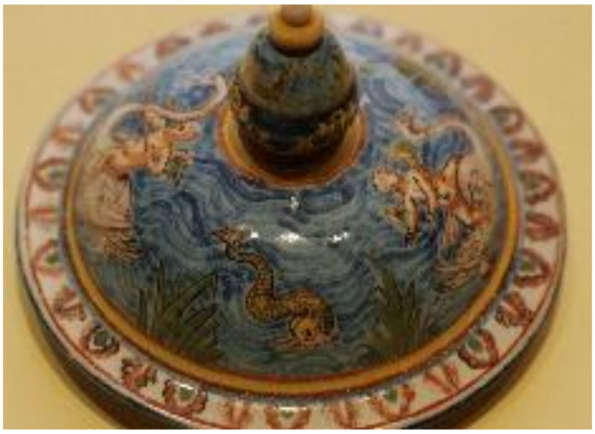

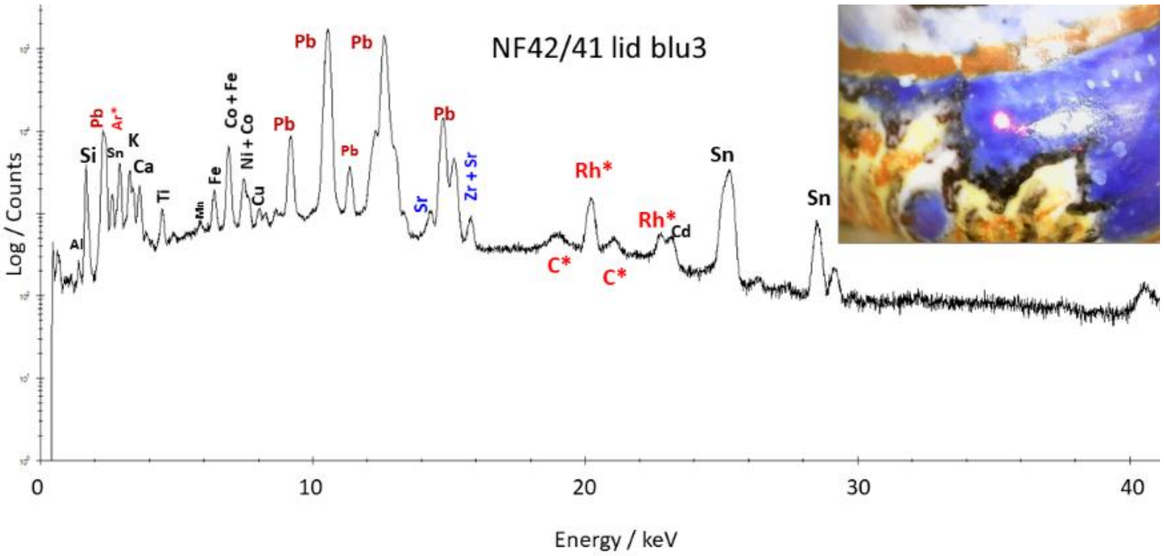

Inv. MNC 27838, *'L'arbre d'amour'* bowl (d: 31.3 cm), 1765

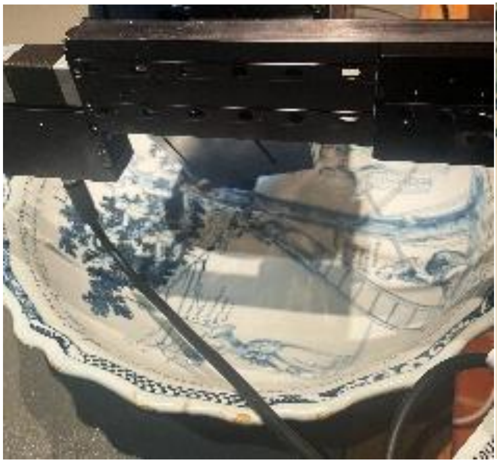

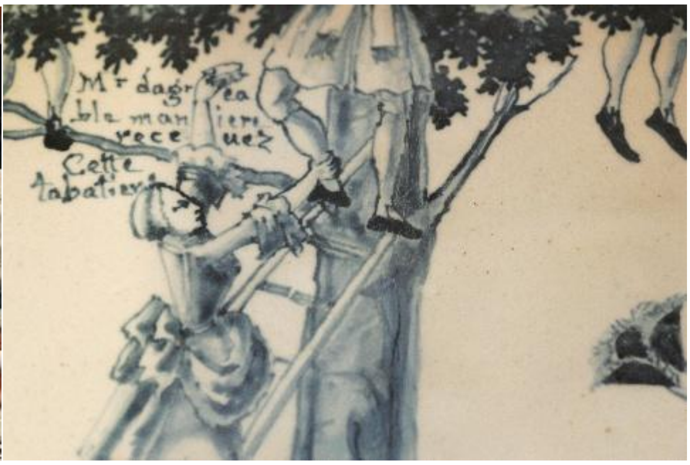

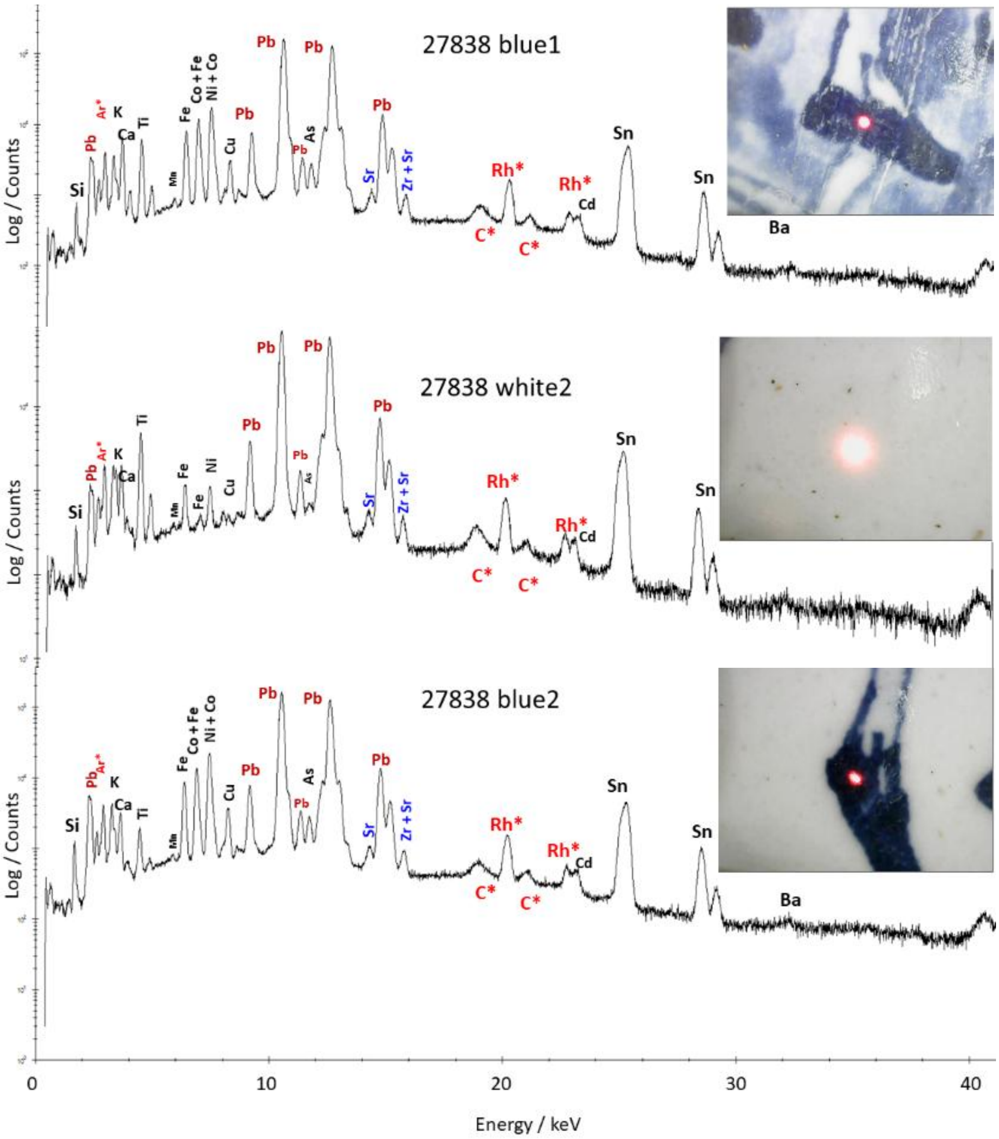

Inv. NF 860, Hanap (15.5 cm), 1792

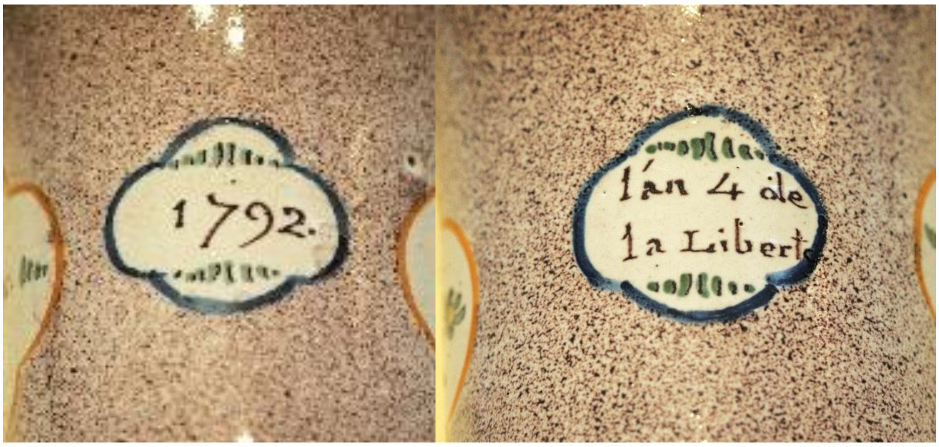

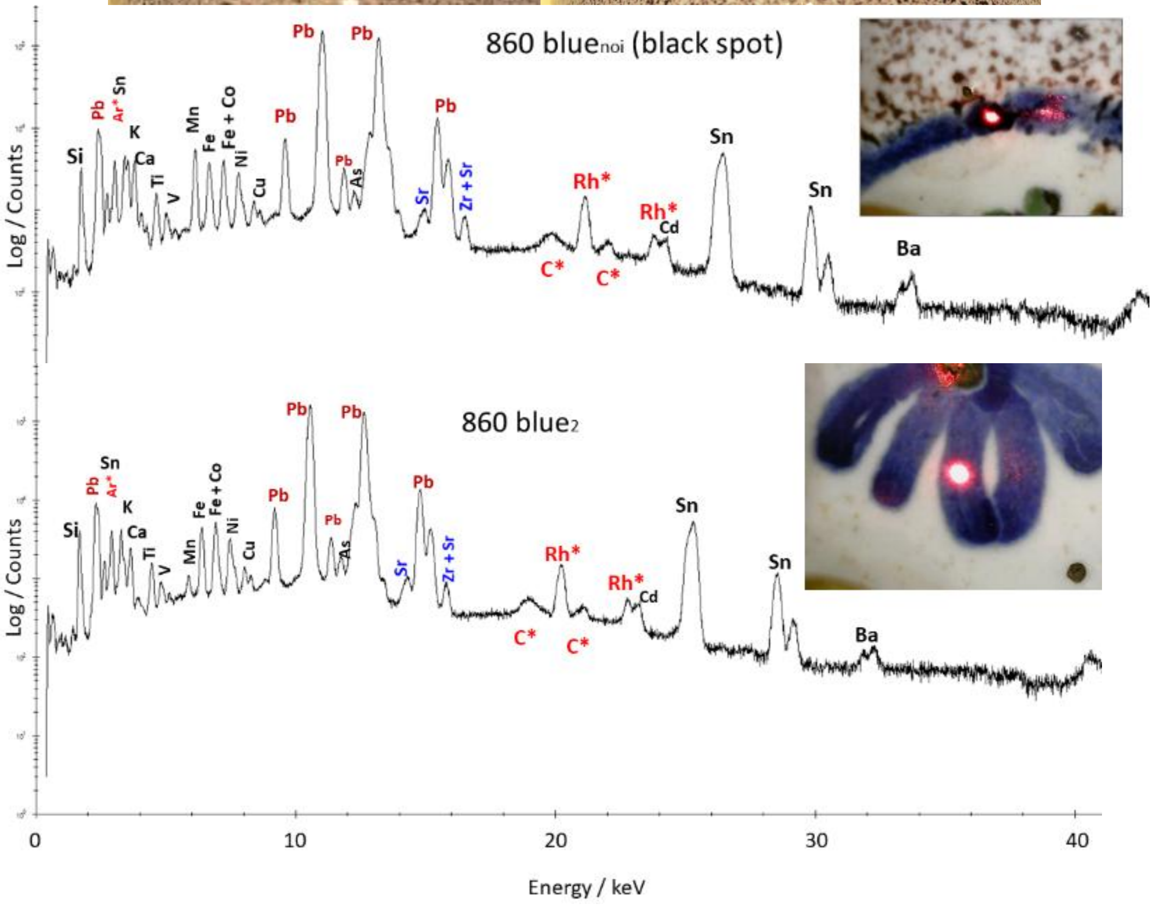

Inv. NF 726, Plate (22.5 cm), 1792

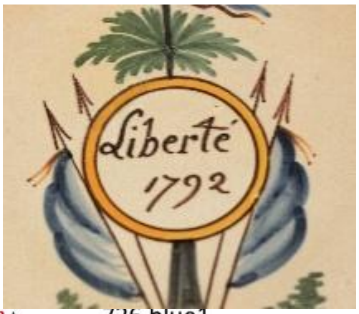

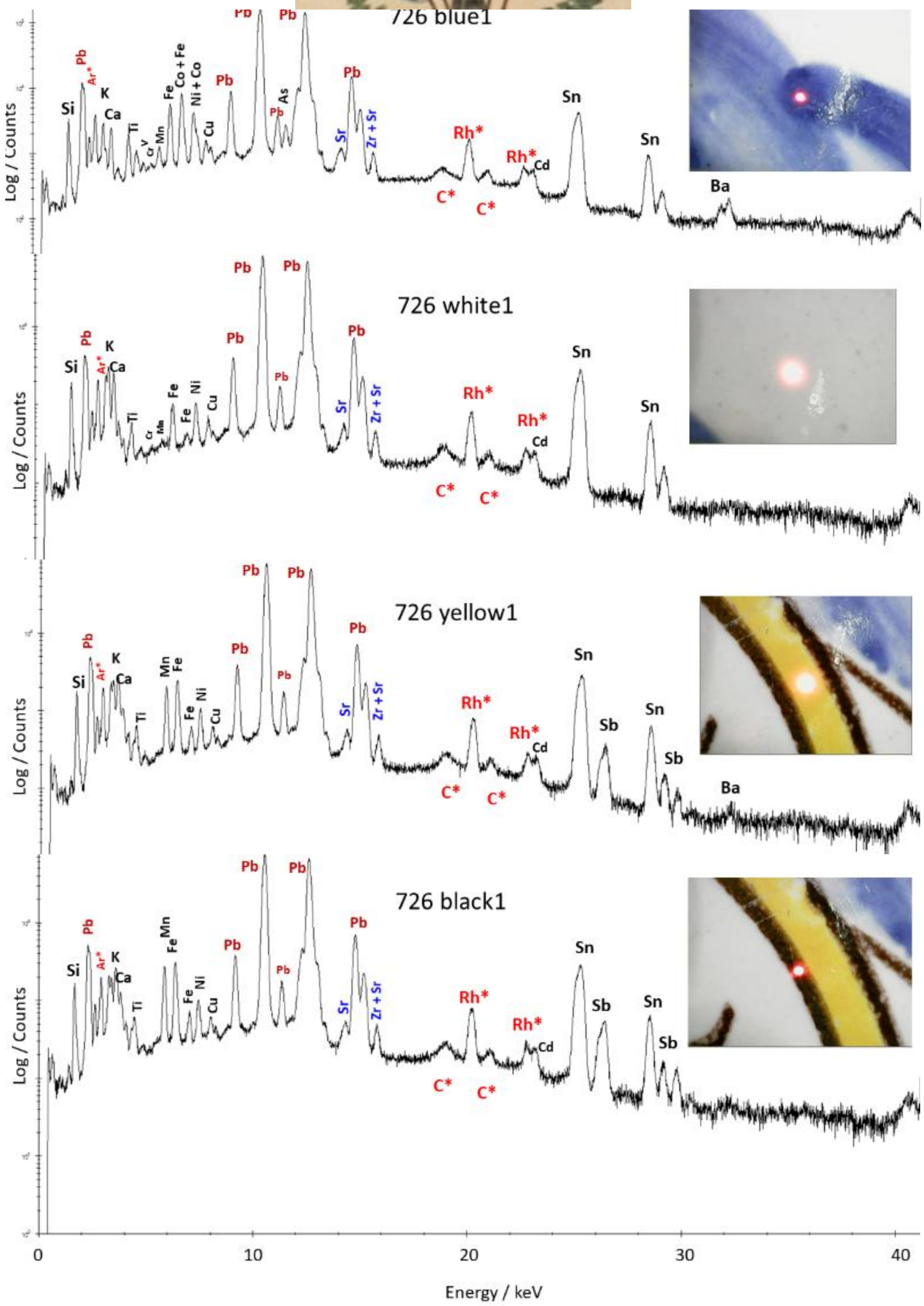

Inv. NF 881 Ewer (h: 34.5 cm), 1805

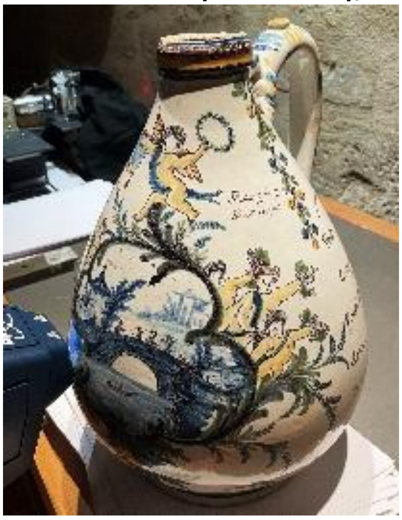

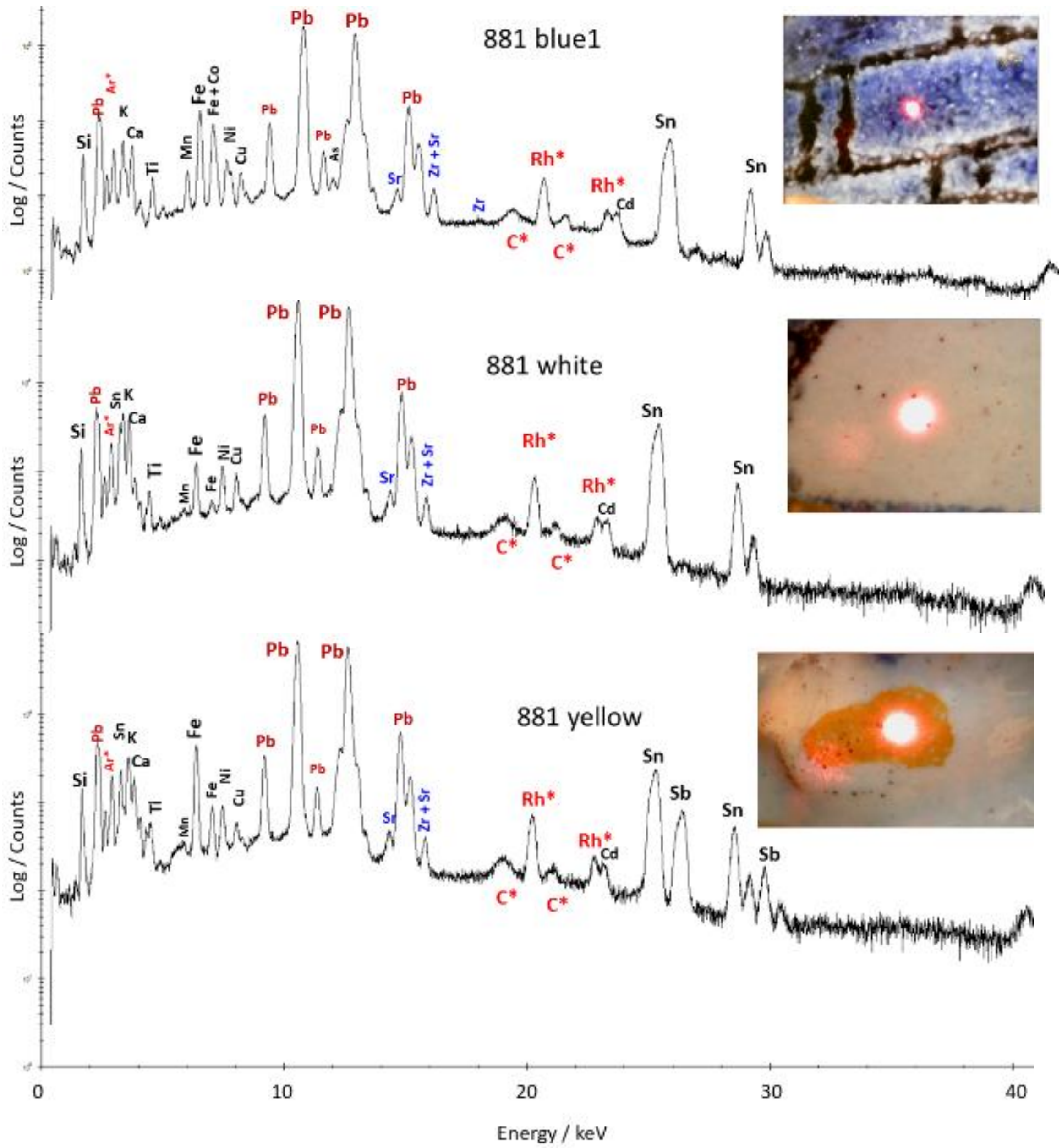

Inv. NF 935, Plate (d: 22.5 cm), 1810

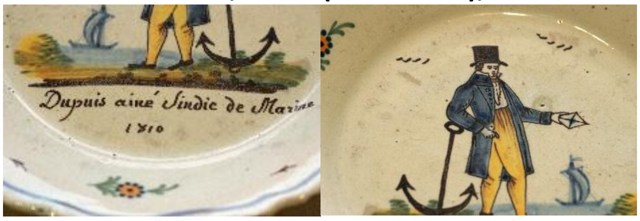

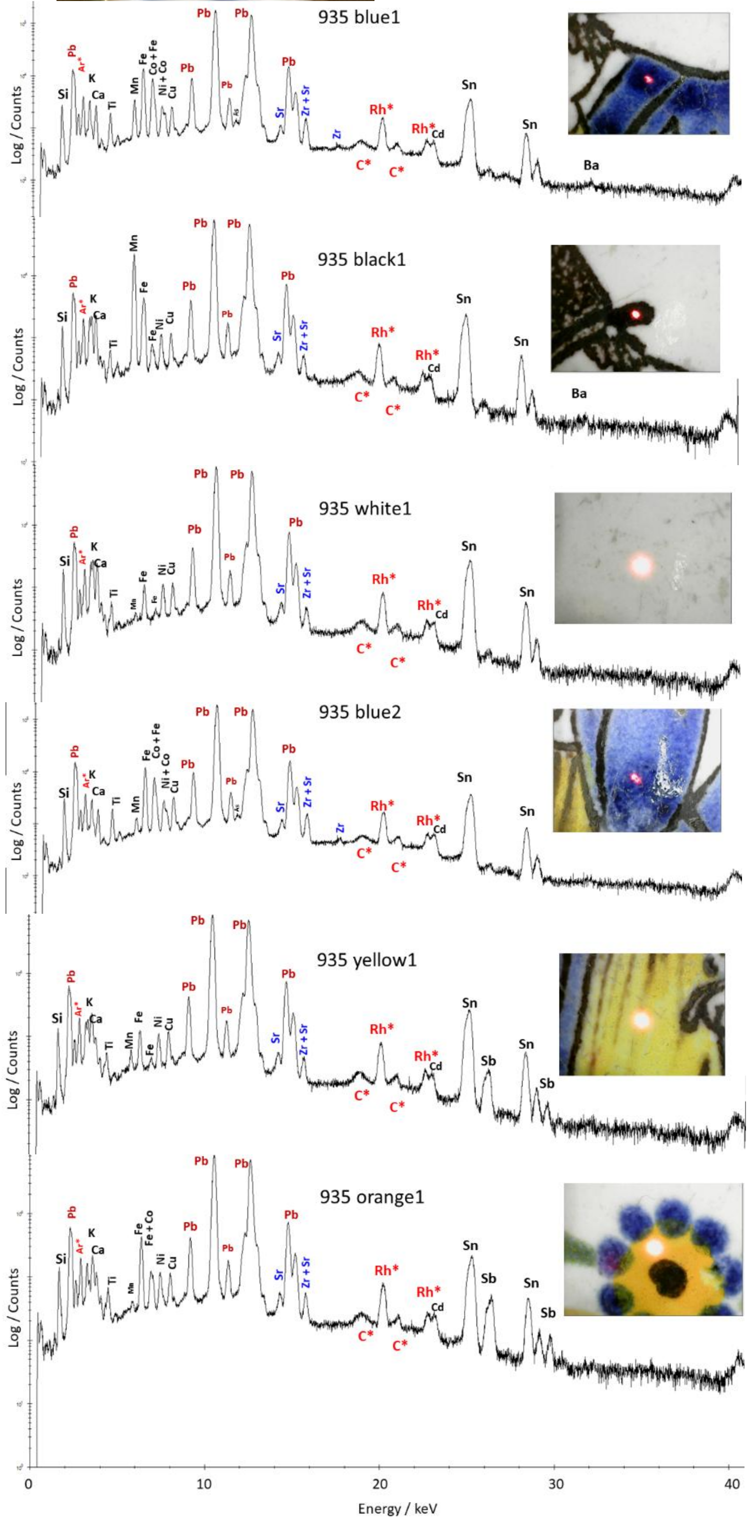

Inv. NF 2017.30.1, Decorate plate (Bacchus)(d: 33 cm), ca. 1825

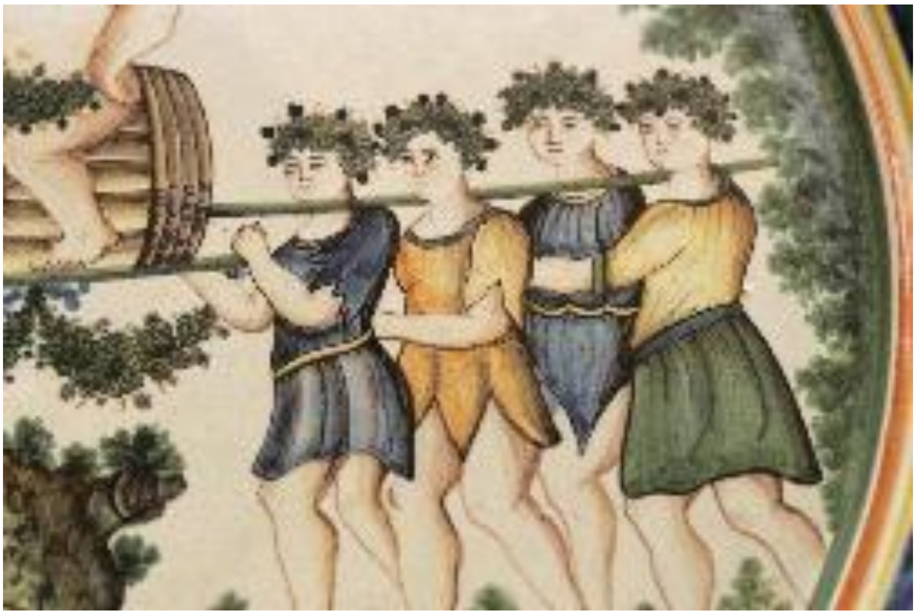

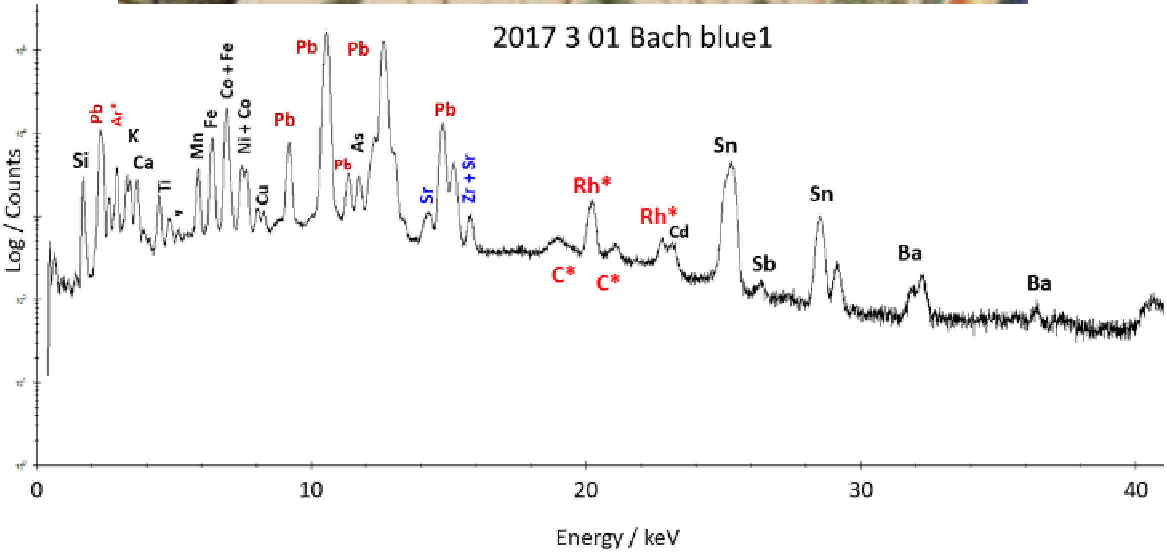

Inv. NF 2017.10.3, Decorate plate (Ruins) (d: 33 cm), ca. 1822

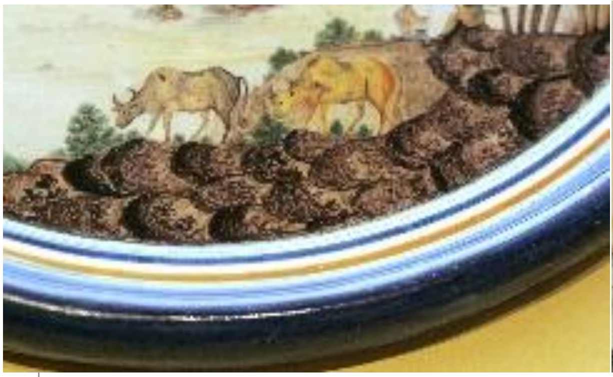

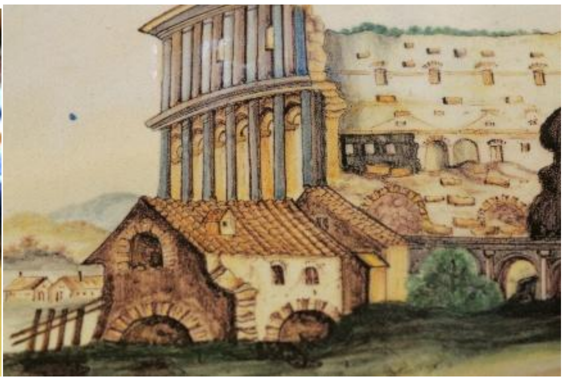

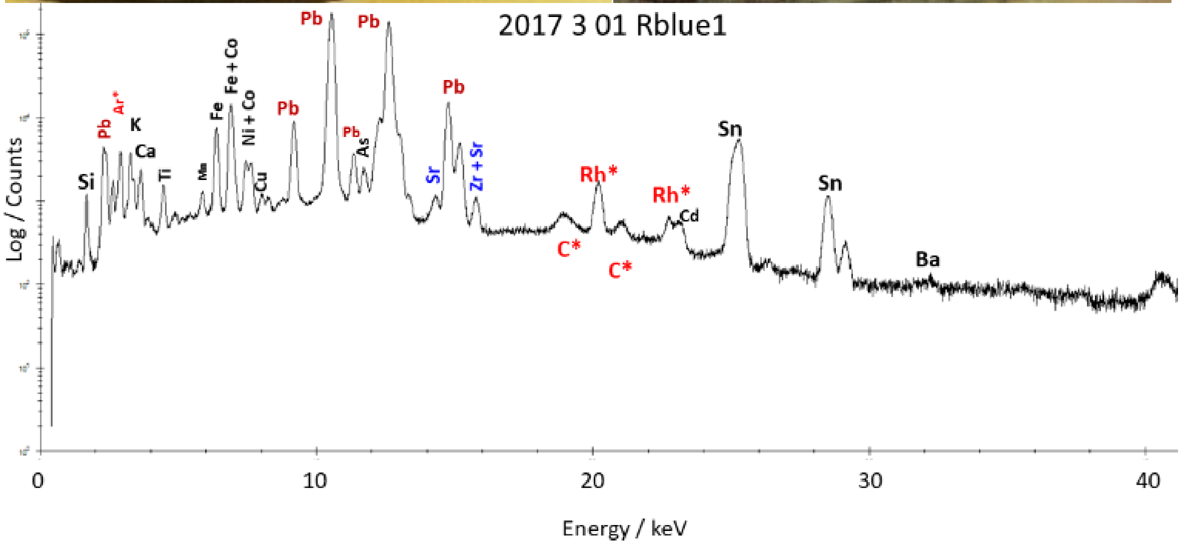

Inv. NF 828, Giraffe plate,(d: 22 cm), ca.1828

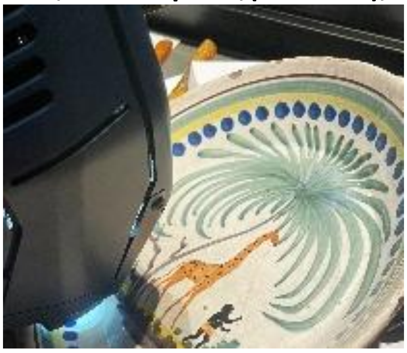

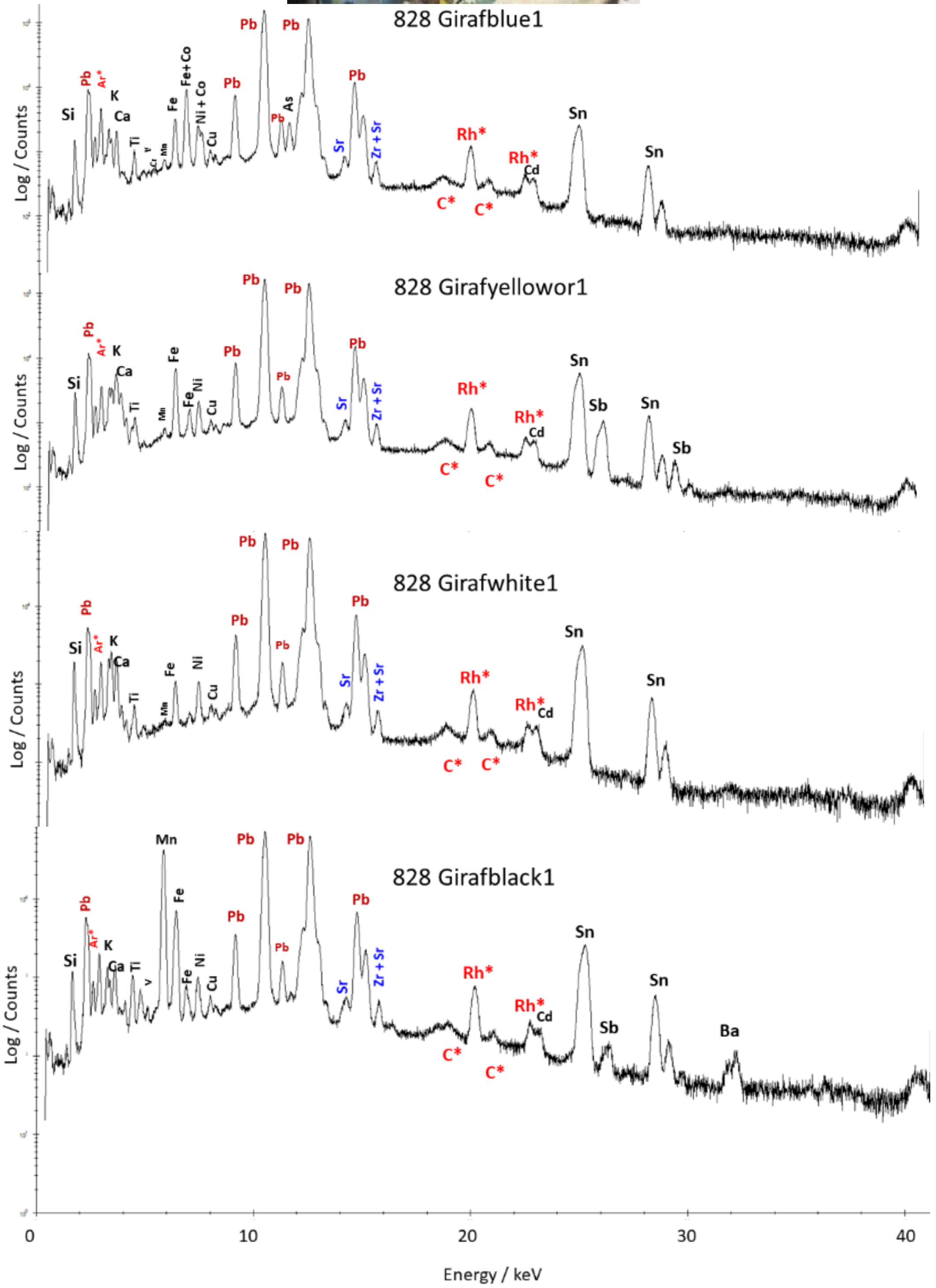

Inv. NF 96511, Plate (d:20 cm), 1836

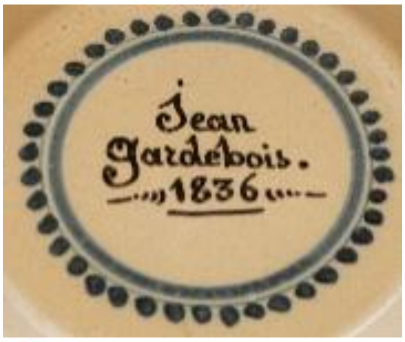

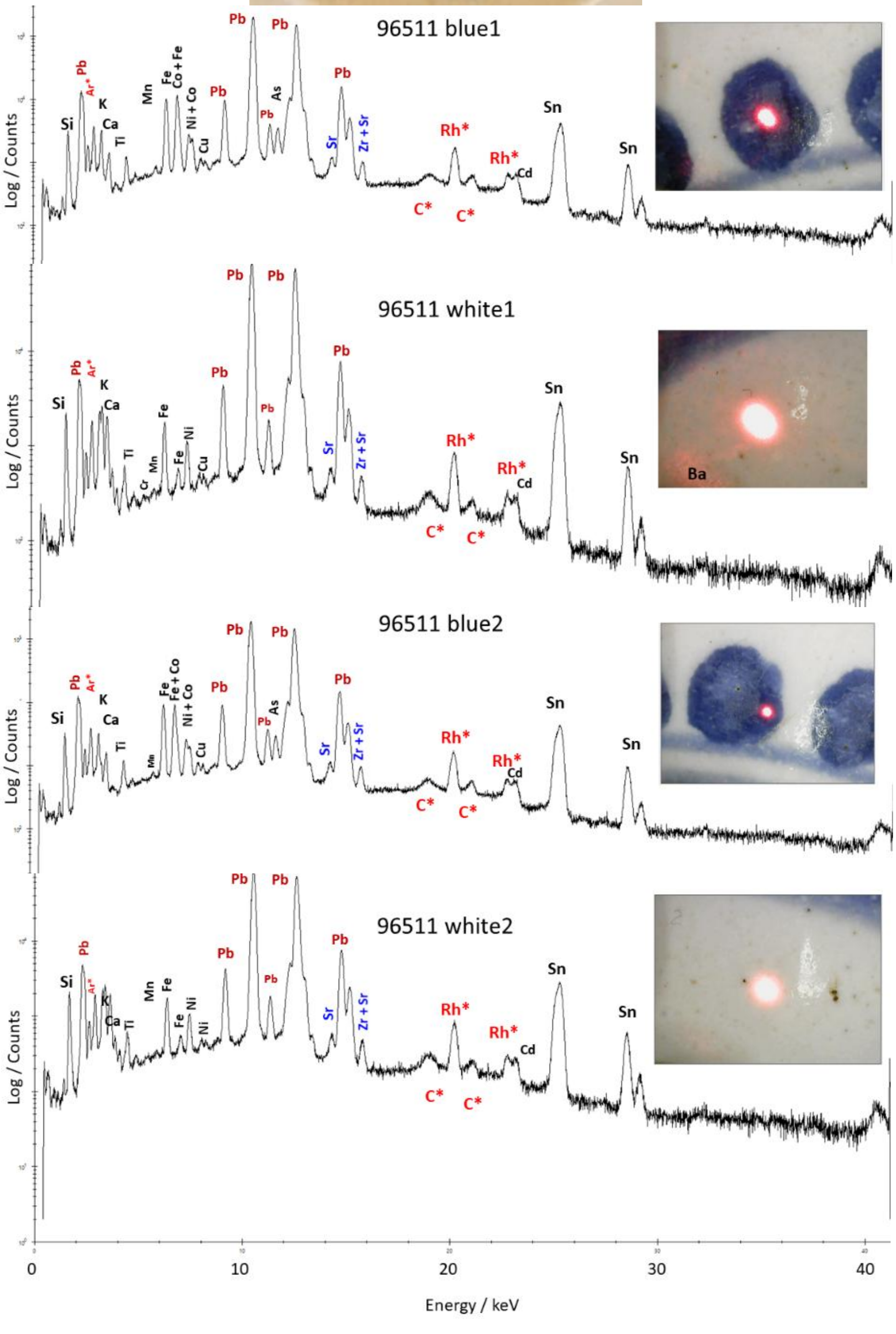

Inv. NF 1066.8 (Ben), Fontaine (h: 44.6 cm), 1845

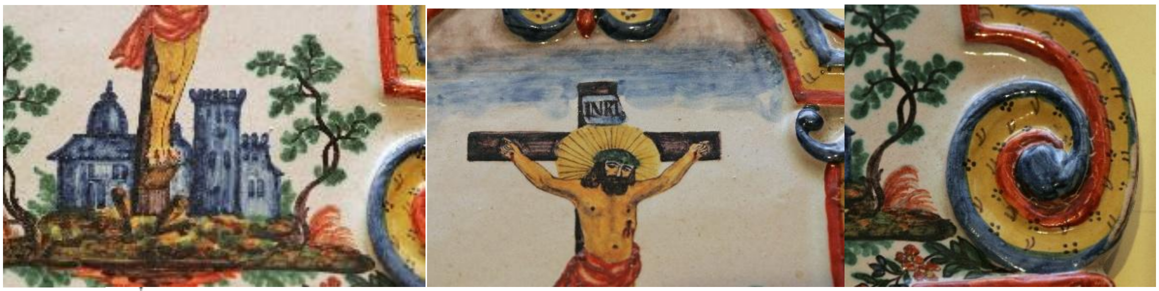

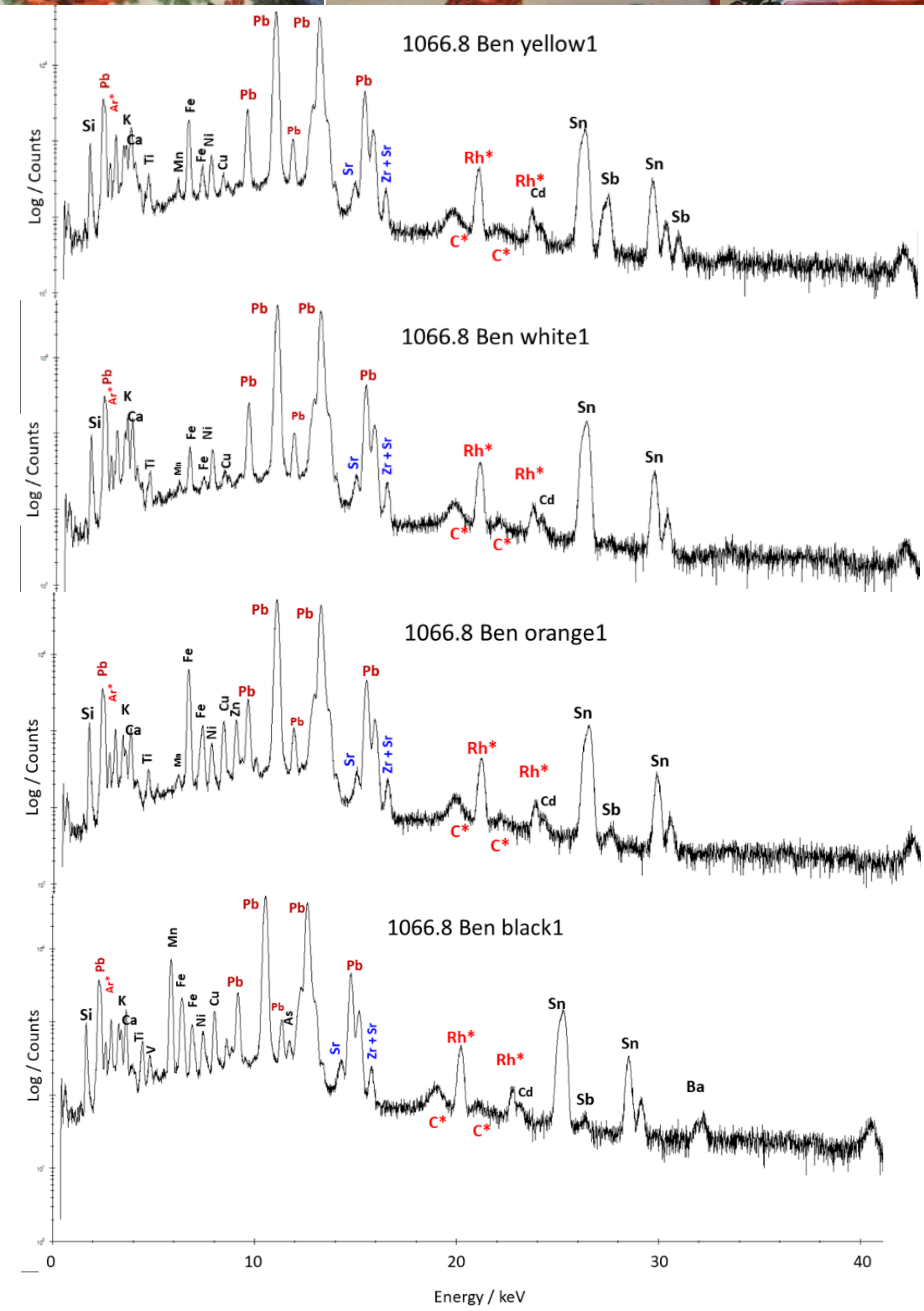

Supplement: Supplementary file 1 [file materials-19-02442-s001.zip › materials-4316168-supplementary/Fig S1_Supplement Materials XRF Faiences from Nevers - Blue, White, Yellow & Black areas.pdf]
