# Supplementary material for: Nevers City Earthenware Blue Glaze: pXRF Categorization from Cobalt Sources and Raw Materials Impurities: Comparison of Reasoned and Chemometrics Methods
Source: Materials (Basel). 2026 Jun 7;19(12):2442. doi: 10.3390/ma19122442 (PMC13301694; doi:10.3390/ma19122442)
Supplement: Supplementary file 1 [file materials-19-02442-s001.zip › materials-4316168-supplementary/Fig. S2 Supplementary Material_Artax procedure.pdf]

Nevers city earthenware blue glaze: pXRF categorization from cobalt sources and raw materials impurities. Comparison of reasoned and chemometrics methods.

Gulsu Simsek-Franci[1], Philippe Colomban[2]*, Marie-Lys Chevalier[3]

[1] Yildiz Technical University, Faculty of Chemical and Metallurgical Engineering, Department of Metallurgical and Materials Engineering, Davutpasa Mah. Davutpasa Caddesi 34220 Esenler - Istanbul, Türkiye,

gulsu.simsek@yildiz.edu.tr

[2] Laboratoire 'De la Molécule au Nano-objet : Réactivité, Interaction et Spectroscopies, (MONARIS UMR8233), Sorbonne Université, CNRS, Campus P.-et-M. Curie, 4 Place Jussieu, 75005 Paris, France ;

philippe.colomban@sorbonne-universite.fr

[3] Musée de la faïence et des Beaux-Arts- 'Frédéric Blandin', 16 rue Saint-Genest, Nevers, France

*Correspondence: philippe.colomban@sorbonne-universite.fr

Figure S2

Summary of steps for processing XRF data files with Bruker Spectra Artax software (version 7.4.0.0)

This evaluation procedure which was developed by Dr. Bruce J. Kaiser (Emeritus Chief Scientist of Bruker AXS), reveals the net number of photons shown in the Data Table of Supplementary Materials. He described all the steps in his instruction notes.

Now open Artax and open your Spectra in Artax

- Click on File and open spectra
- Make sure files of type is on txt
- you can only have 100 spectra on the display at any one time, but you can have up to 4000000 spectra in your Points folder that you do the analysis on.
- So open 100 spectra or less and then add them to the Points folder. Then clear all the spectra on the display and open the second set of 100 or less and click on add spectrum to add those 100 to the Points folder and then clear all the spectra on the display and open the next group and add them to the Points folder.
- Whatever is on the display can be added to the Points folder so you do this cycle until all your spectra are in the Points folder.
- Then you do your analysis on the Points folder.

Using Artax for qualitative analysis

1. Read in your pdz files from the S1PXRF data up to 100 of them at a time
2. Open ARTAX and make a new project

Using Artax for qualitative analysis

1. Create txt files in S1PXRF
2. Open ARTAX and make a new project
3. Choose elements

Using Artax for qualitative analysis

1. Create txt files in S1PXRF
2. Open ARTAX and make a new project
3. Choose elements
4. Create Methods file

Using Artax for qualitative analysis

1. Create txt files in S1PXRF
2. Open ARTAX and make a new project
3. Choose elements
4. Create Methods file
5. Check the method!
6. Deconvolution of spectrum
   - Export results

Using Artax for qualitative analysis

1. Create txt files in S1PXRF
2. Open ARTAX and make a new project
3. Choose elements
4. Create Methods file
5. Check the method!
6. Deconvolution of spectrum
   - Export results

From the Results tab, we are retrieving the number of photons listed under the column “Net”.
In the supplementary material, the number of photons is listed for each element. The following procedures (e.g., normalization by Rh, z-score analysis, etc.) were added in the text.
